# Identification of a circRNA-miRNA-mRNA regulatory network for exploring novel therapeutic options for glioma

**DOI:** 10.7717/peerj.11894

**Published:** 2021-08-06

**Authors:** Yi He, Yihong Chen, Yuxin Tong, Wenyong Long, Qing Liu

**Affiliations:** 1 Neurosurgery Department, Xiangya Hospital Central South University, Changsha, Hunan, China; 2 Department of Ophthalmology, Second Xiangya Hospital, Central South University, Changsha, Hunan, China; 3 Hunan Clinical Research Center of Ophthalmic Disease, Changsha, Hunan, China

**Keywords:** Glioma, circRNA, ceRNA network, Novel therapy, Biomarker

## Abstract

**Background:**

Glioma is the most common brain neoplasm with a poor prognosis. Circular RNA (circRNA) and their associated competing endogenous RNA (ceRNA) network play critical roles in the pathogenesis of glioma. However, the alteration of the circRNA-miRNA-mRNA regulatory network and its correlation with glioma therapy haven’t been systematically analyzed.

**Methods:**

With GEO, GEPIA2, circBank, CSCD, CircInteractome, mirWalk 2.0, and mirDIP 4.1, we constructed a circRNA–miRNA–mRNA network in glioma. LASSO regression and multivariate Cox regression analysis established a hub mRNA signature to assess the prognosis. GSVA was used to estimate the immune infiltration level. Potential anti-glioma drugs were forecasted using the cMap database and evaluated with GSEA using GEO data.

**Results:**

A ceRNA network of seven circRNAs (hsa_circ_0030788/0034182/0000227/ 0018086/0000229/0036592/0002765), 15 miRNAs(hsa-miR-1200/1205/1248/ 1303/3925-5p/5693/581/586/599/607/640/647/6867-5p/767-3p/935), and 46 mRNAs (including 11 hub genes of ARHGAP11A, DRP2, HNRNPA3, IGFBP5, IP6K2, KLF10, KPNA4, NRP2, PAIP1, RCN1, and SEMA5A) was constructed. Functional enrichment showed they influenced majority of the hallmarks of tumors. Eleven hub genes were proven to be decent prognostic signatures for glioma in both TCGA and CGGA datasets. Forty-six LASSO regression significant genes were closely related to immune infiltration. Finally, five compounds (fulvestrant, tanespimycin, mifepristone, tretinoin, and harman) were predicted as potential treatments for glioma. Among them, mifepristone and tretinoin were proven to inhibit the cell cycle and DNA repair in glioma.

**Conclusion:**

This study highlights the potential pathogenesis of the circRNA-miRNA-mRNA regulatory network and identifies novel therapeutic options for glioma.

## Introduction

Gliomas comprise majority of primary intracranial neoplasms with high heterogeneity and aggressiveness, resulting in a poor prognosis even after current standard combination treatments (Ostrom et al., 2018b; Wesseling & Capper, 2018). The five-year survival rate is only approximately 5% (Ostrom et al., 2018a; Ostrom et al., 2018b; Wesseling & Capper, 2018). Recent advances in precision medicine, genomics, immunology, and other disciplines have uncovered multiple experimental therapies, such as targeted therapy, gene therapy, immunotherapy, and novel drug-delivery technologies that could possibly shed light on the treatment strategies for gliomas (Lapointe, Perry & Butowski, 2018). Therefore, it is of great importance to explore the internal mechanisms of gliomas to identify new therapeutic targets.

Circular RNA (circRNA) is a type of non-coding RNA derived from the exon or intron region of a gene (Kristensen et al., 2019). Since there is no 5–3 polarity and polyA tail, circRNAs are more stable than linear RNAs (Kristensen et al., 2019; Meng et al., 2017). CircRNA regulates the expression of a series of genes by modulating every stage of mRNA metabolism, including sequestration of microRNAs (miRNA) or proteins, modulation of transcription, interference with splicing, and translation to produce polypeptides (Chen, 2020; Wu et al., 2020). During the sequestration of miRNAs, circRNAs act as molecular sponges for miRNAs through their miRNA response elements (MREs), thereby de-repressing all target genes of the respective miRNA family (Hansen et al., 2013). Recently, circRNAs have been found to participate in multiple tumor phenotypes, including proliferation, invasion, metabolism, and immune response, making them promising diagnostic and prognostic markers as well as therapeutic targets for cancers (Chen, 2020).

Recent studies have shown that instead of pure malignant cells, the core tumors are actually surrounded by a complex microenvironment, including the immune cells (Binnewies et al., 2018). Immune cells infiltrating the tumor microenvironment have been confirmed with the ability to predict the patients’ clinical outcomes and also the efficacy of immunotherapy. Therefore, identifying immune cells infiltration, especially their pattern correlated with specific genes and gene signatures, is of great significance for estimation of the prognosis of GBM patients and the value of various therapies (Ali et al., 2016; Quail & Joyce, 2013).

**Figure 1:**
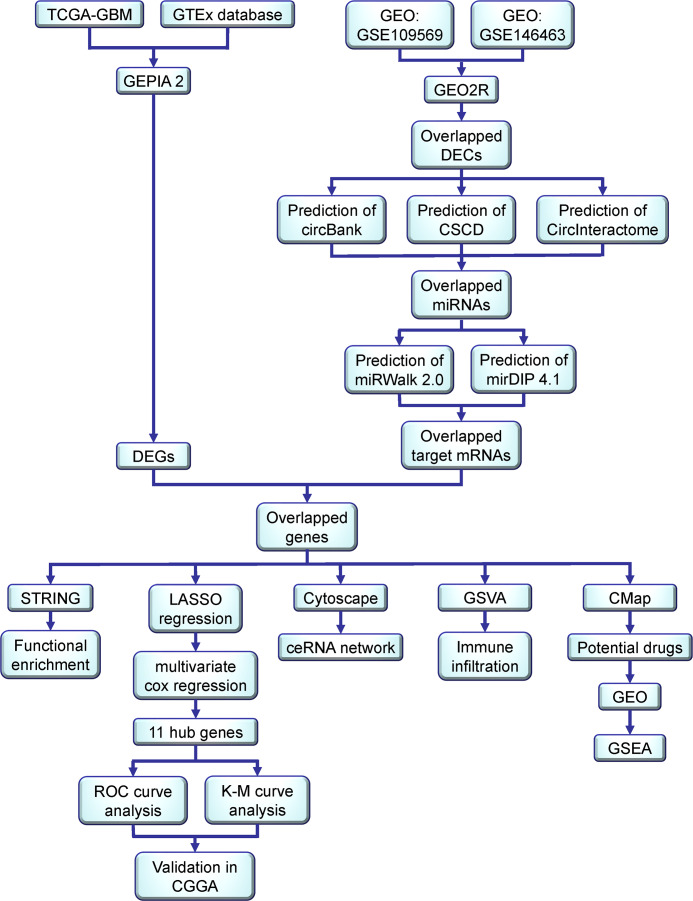
Workflow diagram of the construction of a circRNA-associated ceRNA network, identification and evaluation of a hub gene signature, and the prediction of potential therapeutic options for glioma.

Here, by utilizing bioinformatics methods, we identified differentially expressed circRNAs (DECs) in gliomas and studied their functions in gliomas as competing endogenous RNAs (ceRNAs). The workflow diagram is shown in Fig. 1, where DECs were first acquired from the circRNA-related microarray datasets of gliomas in the GEO database. Then, we forecasted and collected their related miRNAs and their corresponding target genes and built a circRNA-miRNA-mRNA regulatory network. Functional enrichment analyses were performed to determine their potential roles in the pathogenesis of gliomas. Furthermore, the hub genes were obtained through LASSO and multivariate Cox regression analyses and evaluated with ROC curve analysis and K-M curve analysis. Subsequently, GSVA analysis was performed to determine their correlation with immune infiltration. Finally, a connectivity map (CMap) was used to predict corresponding bioactive compounds and potential drugs for treatment, which were further assessed with GSEA in GEO database.

## Materials & Methods

### Data obtained and DECs acquired

Microarray circRNA expression profile data of gliomas and corresponding normal tissues were screened and acquired from the Gene Expression Omnibus (GEO, https://www.ncbi.nlm.nih.gov/geo/) database, which is a public functional genomic database that allows users to query, locate, review, as well as download research and gene expression profiles (Barrett et al., 2013). DECs in the GSE109569 and GSE146463 datasets were analyzed and identified using GEO2R with the criteria of —log2 (fold change)—> 1 and *P* value < 0.05. The circRNAs upregulated or downregulated in both datasets were selected for further analysis.

### Prediction of MREs

We employed three public databases to predict the MREs of the selected DECs: CircBank (http://www.circbank.cn/index.html) is a comprehensive database with more than 140,000 human-annotated circRNAs from different sources, providing abundant information on circRNAs, including their predicted binding miRNAs (Liu et al., 2019). The cancer-specific circRNA database (CSCD, http://gb.whu.edu.cn/CSCD/) is a cancer-specific circRNA database incorporating more than 272,000 cancer-specific circRNAs, with the aim of predicting MRE sites, RNA binding protein (RBP) sites, and open reading frames (ORFs) for each circRNA (Xia et al., 2018). Circular RNA Interactome (CircInteractome, http://circinteractome.nia.nih.gov) is a web tool for mapping RBP and MRE sites on human circRNAs by searching public databases of circRNA, miRNA, and RBP. It has multiple functions, including identifying potential circRNAs that can act as RBP sponges (Dudekula et al., 2016). An overlap in at least two databases was the basis for considering candidate target miRNAs of these DECs used for further mRNA prediction. The regulatory roles of these miRNAs and related regulation pathways were assessed using DIANA-miRPath v3.0 (http://snf-515788.vm.okeanos.grnet.gr/), which is a powerful online software for functional analysis of miRNAs (Vlachos et al., 2015).

### Forecasting miRNA–mRNA interactions

miRNA–mRNA interactions were predicted using two integrated miRNA databases. MiRWalk 2.0 (http://zmf.umm.uni-heidelberg.de/mirwalk2) is a web tool that provides information about validated or putative miRNA–mRNA interactions. For its prediction, 12 algorithms (miRWalk, Microt4, mirbridge Targetscan, RNAhybrid, RNA22, PITA, Pictar2, miRNAMap, miRDB, miRanda, and miRMap) were employed to ensure robustness (Dweep & Gretz, 2015). Here, targeted genes forecasted by at least seven algorithms, along with the validated genes, were selected as candidate genes from miRWalk 2.0. Meanwhile, mirDIP v4.1 (http://ophid.utoronto.ca/mirDIP/) is a miRNA database integrated across 30 different resources, capable of providing nearly 152 million human microRNA–target predictions (Tokar et al., 2018). In the mirDIP v4.1 database, genes predicted by at least 11 algorithms under the very high score class were selected as candidate genes from mirDIP v4.1.

### Obtaining DEGs and overlapped target genes

The Gene Expression Profiling Interactive Analysis (GEPIA) web server is a valuable resource for gene expression analysis based on tumor and normal samples from the TCGA and GTEx databases. GEPIA2 is an updated and enhanced version with higher resolution and more functionalities (Tang et al., 2019). Through GEPIA2, We identified differentially expressed genes (DEGs) between glioblastoma (GBM) and normal tissues using the criteria of —log2 (fold change)—> 1 and *P* value < 0.01. These DEGs were intersected with the candidate gene sets from miRWalk 2.0 and mirDIP v4.1. Overlapped mRNAs showing up in all three sets were taken as final target mRNAs and used for further analysis.

### Functional enrichment analysis of overlapped genes

The Search Tool for the Retrieval of Interacting Genes database (STRING) is a database aimed at achieving a comprehensive and objective global network, including direct (physical) and indirect (functional) interactions (Szklarczyk et al., 2019). It was utilized to perform Gene Ontology (GO) analysis and Kyoto Encyclopedia of Genes and Genomes (KEGG) pathway enrichment analysis for the overlapped mRNA, with a setting *P* < 0.05 and counts > 5.

### Identification and assessment of hub genes

Using mRNA expression profiles and clinical information from TCGA (https://www.cancer.gov/tcga), the overlapping genes were consecutively analyzed with LASSO regression and multivariate Cox regression analysis, and independent prognostic genes were identified as hub genes. The total risk score of each sample was calculated as the sum of the multiplication of the expression value and the correlation coefficient of each gene. Patients with higher 50% or lower 50% of risk score were defined as high-risk or low-risk groups respectively. Their value as a prognostic signature for gliomas, as well as their corresponding sensitivity and specificity, were evaluated using K-M curve analysis and ROC curve analysis both in the TCGA training dataset and the external CGGA validation dataset (http://www.cgga.org.cn/) (Zhao et al., 2021). Sample IDs of the CGGA samples used in this study was listed in Table S4. The protein level expression differences of hub genes were further confirmed with immunohistochemistry (IHC) images from The Human Protein Atlas (HPA) database (Uhlen et al., 2015).

### Construction of a circRNA–miRNA–mRNA network

Cytoscape is an open-source software for the integration of molecular interaction network data and the establishment of powerful visualization (Shannon et al., 2003). Here, it was used to construct a circRNA–miRNA–mRNA regulatory network.

### Assessment of immune cell infiltration

Gene set variation analysis (GSVA) is a gene set enrichment method and an open source software package for R, which can estimate the variation of pathway activity over a sample population in an unsupervised manner (Hanzelmann, Castelo & Guinney, 2013). To estimate the immune cell infiltration level, we applied single-sample gene-set enrichment analysis (ssGSEA), which is a built-in algorithm of the GSVA package, using the RNA-seq data and related clinical data from the TCGA-GBMLGG dataset. Gene expression features of 24 immune cells were acquired from a previous study (Bindea et al., 2013), and the correlation with immune cell infiltration was obtained for the genes that passed LASSO regression analysis. Sample IDs of the TCGA-GBMLGG samples used in this study was listed in Table S5.

### Connectivity Map (CMap) analysis and assessment

The connectivity map (CMap) is a collection of genome-wide transcriptional expression data from human cell lines treated with various drugs or compounds. Functional connections between drugs, genes, and diseases were then uncovered using pattern-matching algorithms and features of common gene expression changes (Lamb, 2007; Lamb et al., 2006). Using the hub genes from multivariate Cox regression analysis, candidate compounds with negative connectivity scores were identified as promising candidate therapeutic approaches. The available related RNA-seq data were acquired from GEO and used for GSEA analysis to explore the effects of those drugs on gliomas.

### Statistical analysis

R software (version 4.0.3) was used for all statistical analyses, and *p*-values <0.05 were considered statistically significant. The Glmnet package and survival package were utilized for LASSO regression and multivariate Cox regression analysis, respectively. GGally and rms packages were used to evaluate and remove the co-linearity between samples. K-M curve analysis was performed using the Survminer package. ROC curve analysis was performed using the survival ROC package. Visualization was achieved using the ggplot2, pheatmap, or plotROC packages.

## Results

### Acquiring eight DECS in gliomas

**Figure 2:**
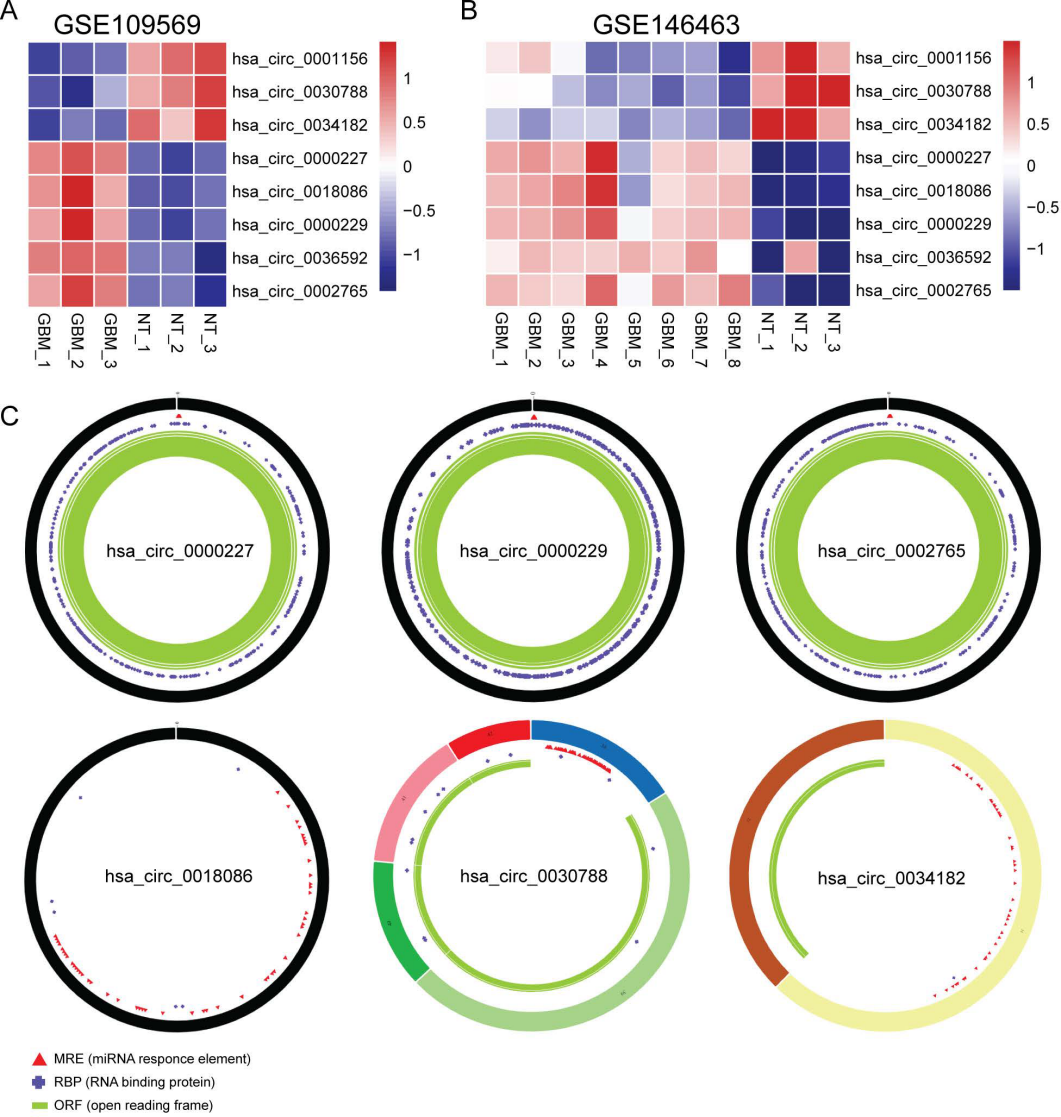
Expression profile heatmaps for 8 DECs in two GEO datasets (A, B) and basic structures of the circRNAs (C). The different colors and shapes in the outer and inner ring represent the different exons and the positions of MRE, RBP and ORF.

To explore the potential function of circRNAs and the corresponding ceRNA network in glioma, DECs from GSE109569 (three glioma samples *vs.* three normal samples) and GSE146463 (eight glioma samples *vs.* three normal samples) datasets were obtained using GEO2R from the GEO database. Genes with *P* < 0.05 and a —Log2(fold change)—> 1 were considered significant DECs. Through the intersection of two DEC datasets, six upregulated and two downregulated circRNAs were identified and chosen as research objects in this study. The differences in expression between gliomas and normal tissues are shown in Figs. 2A–2B. The basic features of these eight circRNAs are listed in Table 1. The circRNAs’ accurate expression values in the GEO datasets are summarized in Table S1.

### Identification of circRNA–miRNA interactions

**Figure 3:**
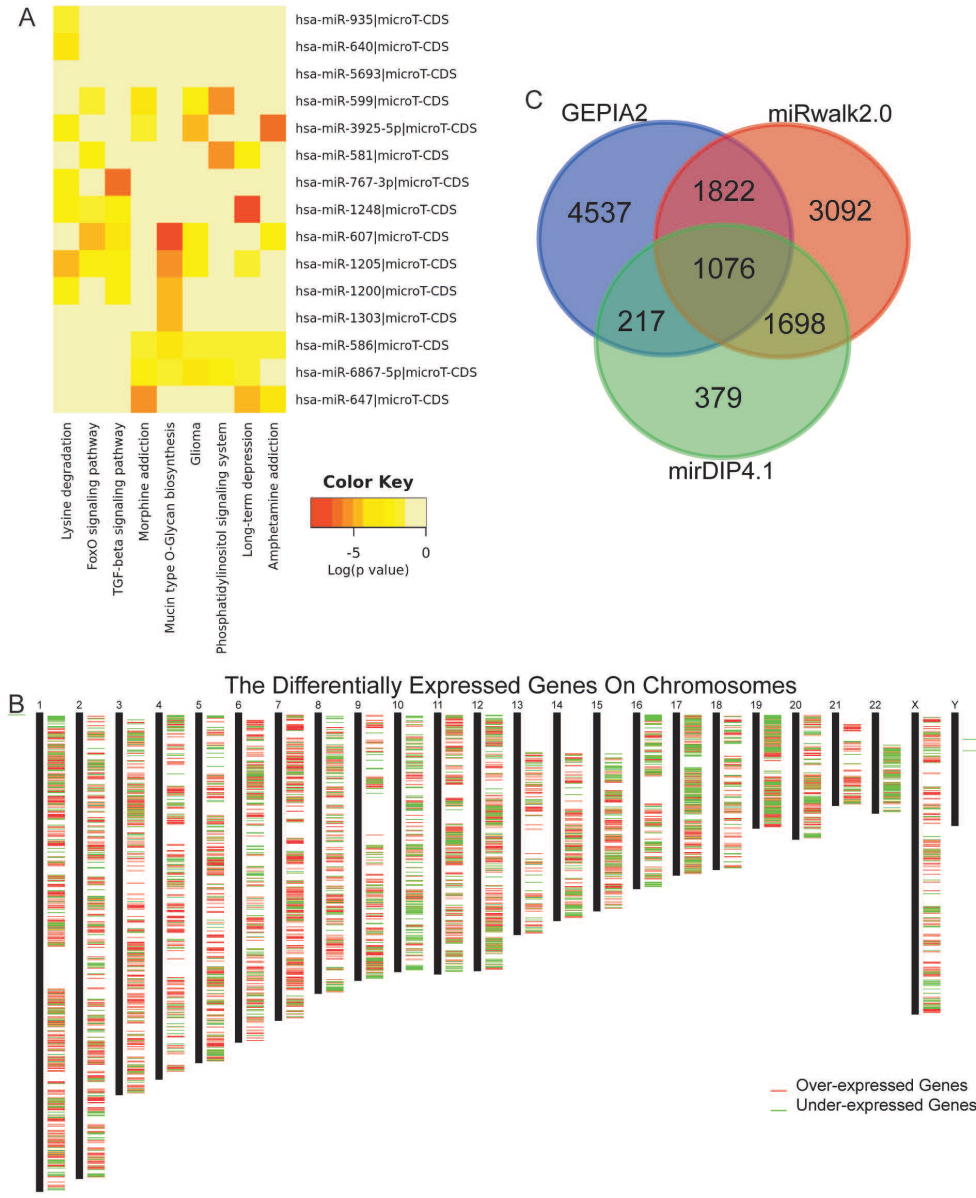
MiRNA pathway enrichment and target genes identification. Significant signaling pathways of the 15 miRNAs utilizing the DIANA-miRPath (A), chromosome distribution of DEGs from GEPIA2 (B), and the Venn graph showing the 1076 overlapped target genes identified with intersection of three gene sets (C).

To explore the roles of these eight circRNAs as ceRNAs in glioma, three online databases, namely circBank, CSCD, and CircInteractome, were utilized to collect potential target miRNAs. Six out of eight circRNAs were recorded in the CSCD database, and their structures of MRE, RBP, and ORF are shown in Fig. 2C. A total of 15 miRNAs and 18 circRNA–miRNA interactions were identified by at least two databases, including hsa_circ_0030788-miR-5693/miR-6867-5p/miR-607/miR-1248/miR-586/miR-599, hsa_circ_0034182-miR-1200, hsa_circ_0000227-miR-647/miR-1303/miR-767-3p, hsa_circ_0018086-miR-1303/miR-3925-5p/miR-581, hsa_circ_0000229-miR-935/miR-640, hsa_circ_0036592-miR-1205/miR-767-3p, and hsa_circ_0002765- miR-587/miR-767-3p. The functions of these miRNAs in tumors reported in PubMed were summarized in Table 2. Among them, miR-1303, miR-581, miR-586, miR-599, miR-607, miR-647, miR-767-3p, miR-935, and miR-1248 have been extensively reported to regulate the progression of various tumors as promoters or suppressors. DIANA-miRPath was then used to probe signaling pathways involving 15 unique miRNAs. As shown in Fig. 3A, these miRNAs are involved in multiple pathways of glioma, including the FoxO signaling pathway, phosphatidylinositol signaling pathway, and TGFβ signaling pathway. Furthermore, given that the expression levels of the miRNAs are critical for their biological functions, we checked the expression levels of those 15 miRNAs in glioma tissues with CGGA data and GEO data, as shown in (Tables S2–S3) Different data sets showed slightly different expression of those miRNAs. And their expression values vary quite a lot from sample to sample. But in general, according to those two datasets, the miR607 and miR587 have relatively lower expression levels.

**Table 1:** Basic features of Differentially expressed circRNAs.

**circBase ID**	**bestTranscript**	**Position**	**strand**	**Length**	**Host gene Symbol**	**circRNA type**	**Regulation**
hsa_circ_0001156	NM_015568	chr20: 37547116-37547282	+	166	PPP1R16B	exonic	down
hsa_circ_0030788	NM_052867	chr13: 101997616-102031004	−	508	NALCN	exonic	down
hsa_circ_0034182	NM_000814	chr15: 26825465-26828561	−	221	GABRB3	exonic	down
hsa_circ_0000227	NM_030751	chr10: 31644072-31676195	+	32123	ZEB1	intronic	up
hsa_circ_0018086	NM_001128128	chr10: 31676052-31676195	+	143	ZEB1	intronic	up
hsa_circ_0000229	NM_030751	chr10: 31661946-31709678	+	47732	ZEB1	intronic	up
hsa_circ_0036592	NR_004859	chr15: 85180577-85181708	+	156	SCAND2	exonic	up
hsa_circ_0002765	NM_001128128	chr10: 31644075-31676727	+	32652	ZEB1	intronic	up

**Table 2:** Functions of miRNAs identified for ceRNA network.

predicted upstream hsa_circ_#	has-miR- #	Tumor type	Regulation axis	Role of miRNA in tumor	Ref.
0000227	1303	Breast Cancer	miR-1303/CDKN1B	Promotor	Chen et al. (2020b)
		Breast Cancer	HIF-1 α/lncRNA-BCRT1/miR-1303/PTBP3	Suppressor	Liang et al. (2020)
		Gastric Cancer	miR-1303/CLDN18	Promotor	Zhang et al. (2014)
		Neuroblastoma	miR-1303/GSK3 β	Promotor	Li et al. (2016)
0018086	581	Colorectal Cancer	miR-581/SMAD7	Promotor	Zhao et al. (2020)
		Hepatocellular Carcinoma	miR-581/EDEM1	Promotor	Wang et al. (2014)
0030788	586	Cervical Cancer, Colon Cancer, etc.	lncRNA-MIF/miR-586	Promotor	Zhang et al. (2016)
		Osteosarcoma	miR-586	Promotor	Yang et al. (2015)
0030788	599	Anaplastic Thyroid Carcinoma	lncRNA-NEAT1/miR-599	Suppressor	Tan et al. (2020)
		Esophageal Carcinoma	circ_0030018/miR-599/ENAH	Suppressor	Wang et al. (2019b)
		Esophageal Carcinoma	HIPK3/miR-599/c-MYC	Suppressor	Ba et al. (2020)
		Gastric Cancer	circ_0008035/miR-599/EIF4A1	Suppressor	Li et al. (2020)
		Gastric Cancer	miR-599/EIF5A2	Suppressor	Wang et al. (2018)
		Hepatocellular Carcinoma	miR-599/MYC	Suppressor	Tian et al. (2016)
		Hepatocellular Carcinoma	circ_0006916/miR-599/SRSF2	Suppressor	Zhu et al. (2020)
		Osteosarcoma	circ_0001721/miR-599	Suppressor	Li et al. (2019)
		Papillary Thyroid Carcinoma	miR-599/Hey2	Suppressor	Wang et al. (2020)
0030788	607	Cervical Cancer	LncRNA-TP73-AS1/miR-607/CCND2	Suppressor	Zhang et al. (2019)
		Chronic Lymphocytic Leukemia	circ-CBFB/miR-607 /FZD3	Suppressor	Xia et al. (2018)
		Lung Squamous Carcinoma Cells	miR-607/CANT1	Suppressor	Qiao et al. (2021)
		Osteosarcoma	LINC00607/miR-607/E2F6	Suppressor	Zheng et al. (2020)
		Pancreatic Cancer	LINC01559/miR-607/YAP	Suppressor	Lou et al. (2020)
		Prostate Cancer	miR-607/BLM	Suppressor	Chen et al. (2019)
0000227	647	Cervical Cancer	LncRNA-ZNFX1-AS1/miR-647	Suppressor	Yang et al. (2020)
		Colorectal Cancer	miR-647/NFIX	Promotor	Liu et al. (2017a)
		Gastric Cancer	miR-647/TP73	Promotor	Zhang et al. (2018a)
		Gastric Cancer	miR-647/ANK2, FAK, MMP2, MMP12, CD44, SNAIL1	Suppressor	Cao et al. (2017)
		Gastric Cancer	miR-647/SRF/MYH9	Suppressor	Ye et al. (2017)
		Gastric Cancer	LncRNA-PROX1-AS1	Suppressor	Song, Bi & Guo (2019)
		Gastric Cancer	miR-647/ANK2	Suppressor	Cao et al. (2018)
		Glioma	miR-647/HOXA9	Suppressor	Qin et al. (2020)
		Non-Small Cell Lung Cancer	miR-647/IGF2	Suppressor	Jiang, Zhao & Yang (2021)
		Non-Small Cell Lung Cancer	miR-647/TRAF2	Suppressor	Zhang et al. (2018b)
		Osteosarcoma	circ_0001649/miR-647	Promotor	Sun & Zhu (2020)
		Ovarian Cancer	circ-FAm53B/miR-647/MDM2	Suppressor	Sun, Liu & Zhou (2019)
		Prostate Cancer	NF-KappaB/circNOLC1/miR-647/PAQR4	Suppressor	Chen et al. (2020a)
0000227, 0036592	767-3p	Glioma	miR-767-3p	Suppressor	Kreth et al. (2013)
		Hepatocellular Carcinoma	circ_0000673/miR-767-3p/SET	Suppressor	Jiang et al. (2018)
		Lung Adenocarcinoma	miR-767-3p/CLDN18	Suppressor	Wan et al. (2018)
		Non-Small Cell Lung Cancer	circ_0018818/miR-767-3p	Suppressor	Xu et al. (2020)
0000229	935	Gastric Cancer	miR-935/SOX7	Promotor	Yang et al. (2016)
		Gastric Signet Ring Cell Carcinoma	miR-935/Notch1	Suppressor	Yan et al. (2016)
		Glioblastoma	miR-935/FZD6	Suppressor	Zhang et al. (2021)
		Glioma	miR-935/HIF1 α	Suppressor	Huang et al. (2020)
		Non-Small-Cell Lung Cancer	miR-935/SOX7	Suppressor	Peng et al. (2018)
		Non-Small-Cell Lung Cancer	miR-935/E2F7	Suppressor	Wang et al. (2019a)
		Osteosarcoma	miR-935/HMGB1	Suppressor	Liu et al. (2018)
		Liver Cancer	miR-935/SOX7	Promotor	Liu et al. (2017b)
0030788	1248	Non-Small-Cell Lung Cancer	miR-1248/TYMS	Promotor	Xu et al. (2014)

### Obtaining target mRNAs in the ceRNA network

To forecast the target genes of these miRNAs, miRWalk 2.0, mirDIP v4.1, and GEPIA2 were utilized in this study. A total of 7688 mRNAs were validated or predicted by at least seven algorithms in miRWalk 2.0. A total of 3370 genes were forecasted by more than 10 algorithms in mirDIP v4.1 to be targets of those miRNAs. In GEPIA2, 7652 genes were identified as significant DEGs with *P* < 0.01 and —Log2(fold change)—> 1. The chromosomal distribution of DEGs is displayed in Fig. 3B. Thereafter, through the intersection of all three gene sets, we obtained a total of 1076 target mRNAs involved in the ceRNA network (Fig. 3C), whose overall expression levels were not significantly correlated with genders (Fig. S1).

### Function enrichment analyses

**Figure 4:**
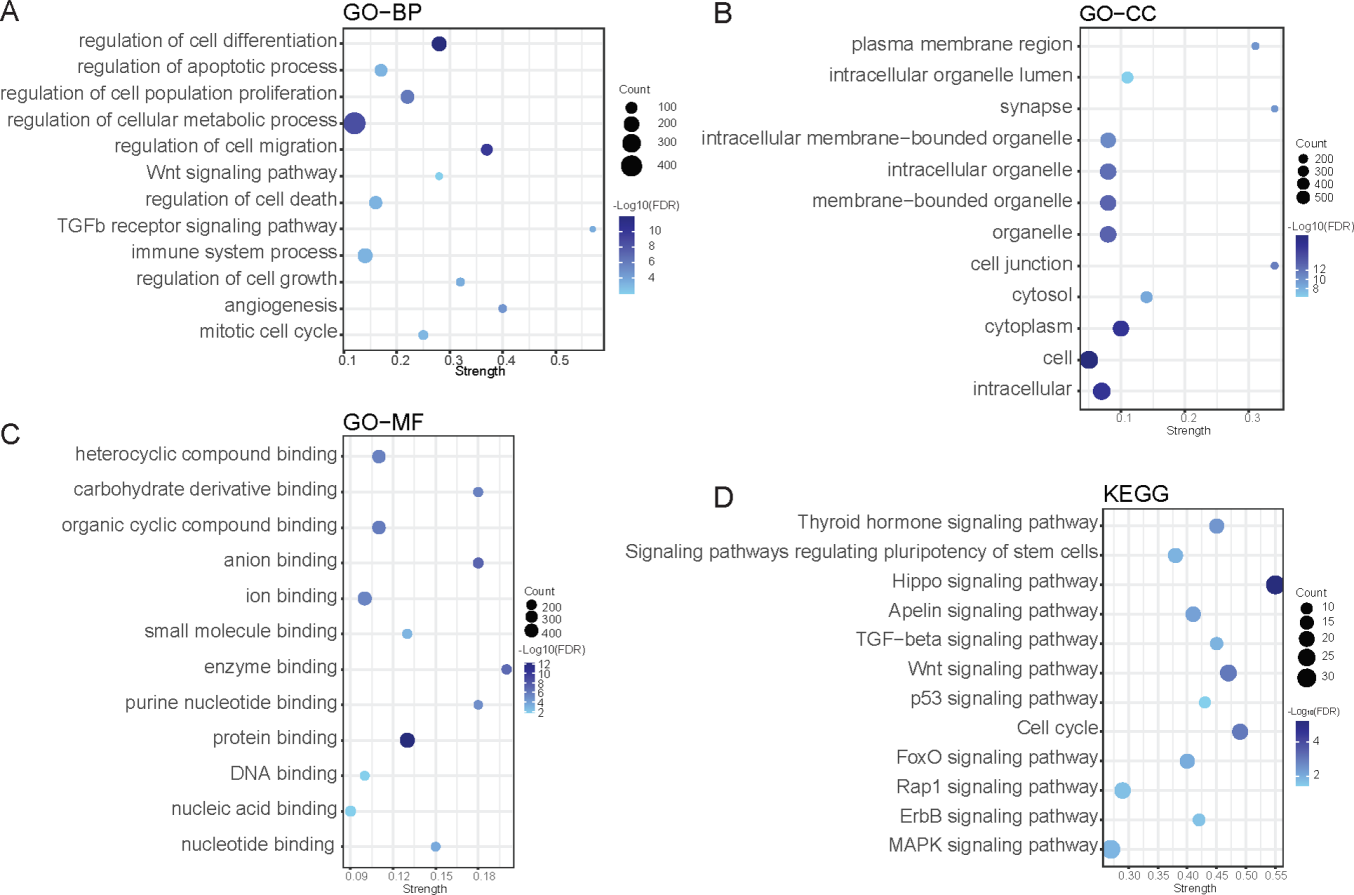
Functional enrichment analysis of target mRNAs in genes sets of biological process (A), cellular component (B), molecular function (C), and KEGG pathways (D). The color intensity of the nodes shows the degree of enrichment of this analysis. Strength is the ratio between observed counts and the expected matching counts for a random list of the same size. The dot size represents the count of genes in a pathway.

To explore the potential functions of the 1076 target genes, GO and KEGG signal pathway enrichment analyses were performed using STRING. In terms of biological processes, these target genes covered majority of the hallmark pathways of tumors, including cell growth, cell cycle, proliferation, differentiation, migration, apoptosis, cell death, immune response, angiogenesis, as well as some well-known cancer-related pathways such as the Wnt and TGFβ signaling pathways, which were also exhibited in KEGG enrichment results. In addition, KEGG also showed enrichment in some other essential pathways, such as the cell cycle, p53, Hippo, MAPK, and stemness regulating pathways. The compositions of the target genes encompassed a wide range of cellular components and molecular functions from cytosol to synapse as well as from DNA binding to enzyme binding. Visualization of the enrichment results is displayed in Fig. 4. Taken together, functional enrichment results indicated that the ceRNA network is extensively involved in the pathogenesis of gliomas.

### Identification and assessment of hub genes

**Figure 5:**
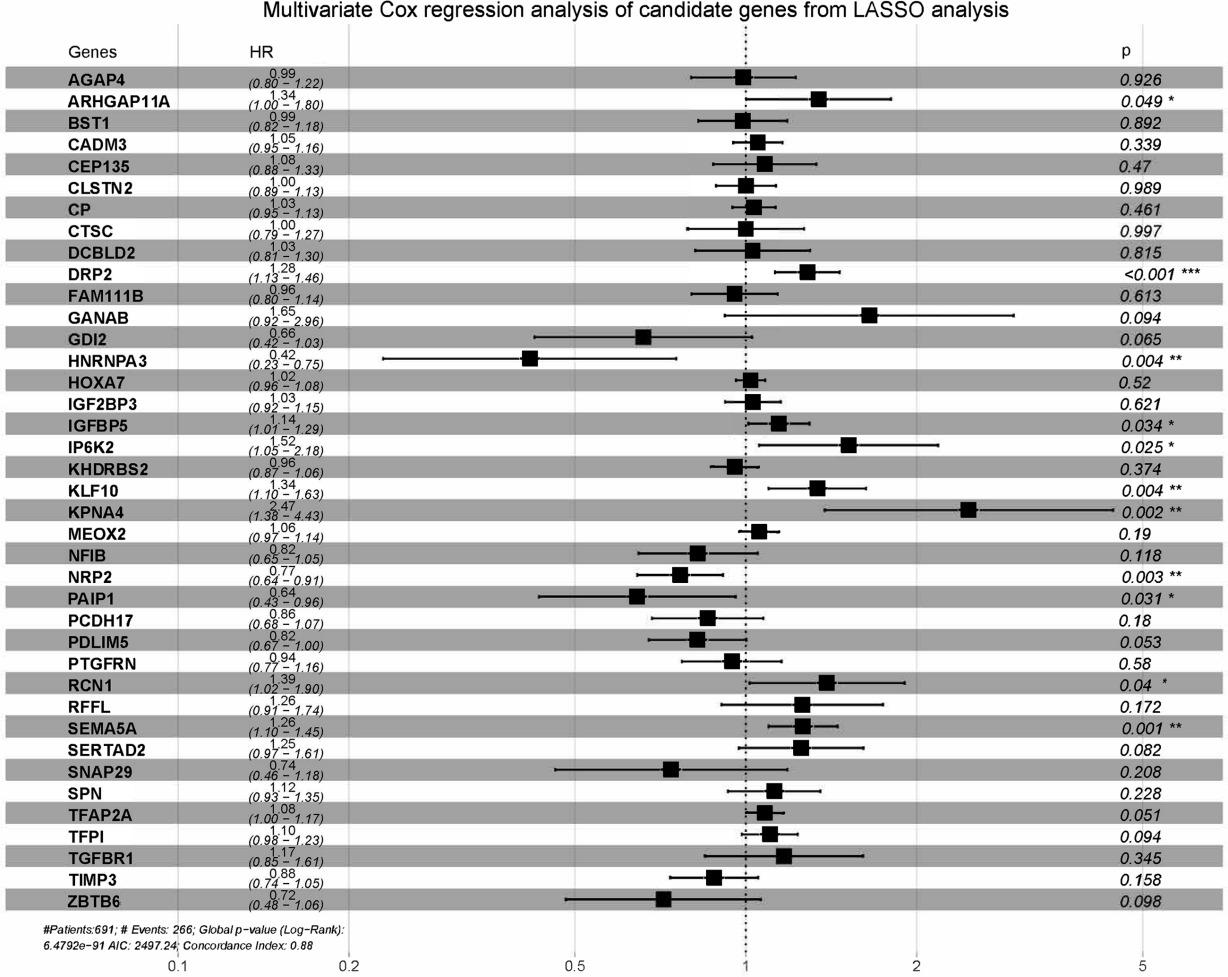
Forest plot of the multivariate Cox regression analysis result of LASSO significant genes utilizing gene expression data and corresponding clinical data from the TCGA-GBMLGG data set. Genes with P < 0.05 were considered significant.

Utilizing gene expression data and corresponding clinical data from the TCGA-GBMLGG data set, we applied LASSO regression analysis to the 1076 target genes and obtained 46 significant candidate genes which were further used for multivariate Cox regression analysis (Fig. 5). Eventually, 11 independent prognosis-related hub genes were identified, including ARHGAP11A, DRP2, HNRNPA3, IGFBP5, IP6K2, KLF10, KPNA4, NRP2, PAIP1, RCN1, and SEMA5A. Thereafter, we checked the human protein atlas database to confirm the protein expression level changes of the 11 hub genes. Three of them (DRP2, IGFBP5, KLF10) are not provided with protein expression information. Two (ARHGAP11A, NRP2) of them showed no big difference between normal and glioma samples. The other six of them (HNRNPA3, IP6K2, KPNA4, PAIP1, RCN1, SEMA5A) showed significantly increased protein expression, consistent with our results (Fig. S2). The genes with supporting protein level results could be of more importance for further future validation study. But given the limited sample size of this part of data, we still took all those 11 genes as our subjects of analysis.

**Figure 6:**
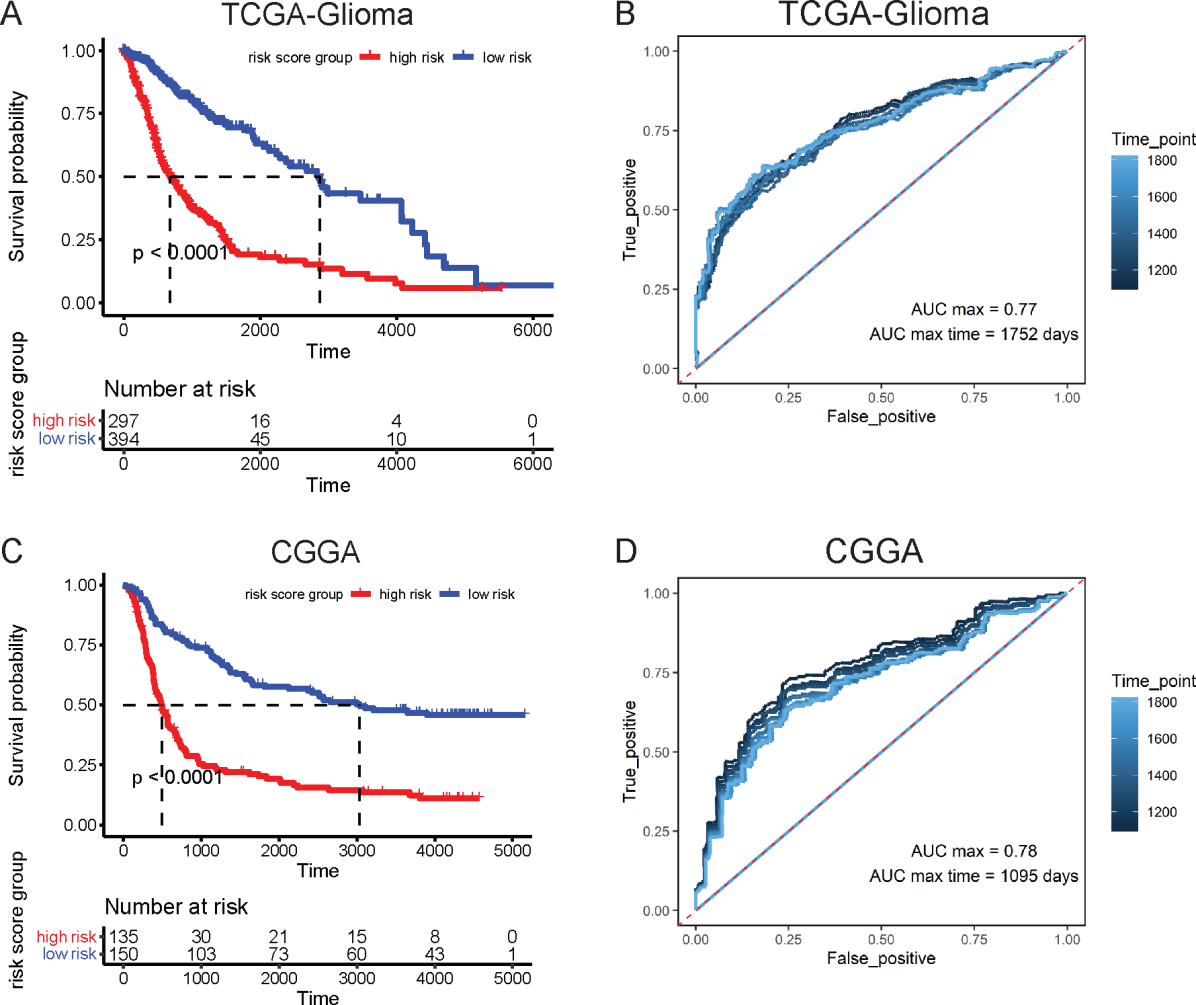
Kaplan–Meier survival curves analysis and ROC curves analysis. Kaplan–Meier survival curves of risk groups in the training dataset (A) and validation dataset (C), as well as the ROC curves of the hub gene signature in the training set (B) and validation set (D).

Based on the expression value and correlation coefficients, we integrated these 11 genes as a whole signature and computed the total risk score of each sample to divide glioma patients into high- and low-risk groups. The K-M curve analysis indicated that the overall survival of the high-risk group was significantly shorter than that of the low-risk group (Fig. 6A). ROC curve analysis further showed decent sensitivity and specificity of this signature in predicting the prognosis of glioma patients (Fig. 6B). An external independent CCGA dataset was introduced to validate the results of the K-M curve and ROC curve analyses, and similar conclusions were obtained (Figs. 6C–6D).

### Construction of a circRNA–miRNA–mRNA network

**Figure 7:**
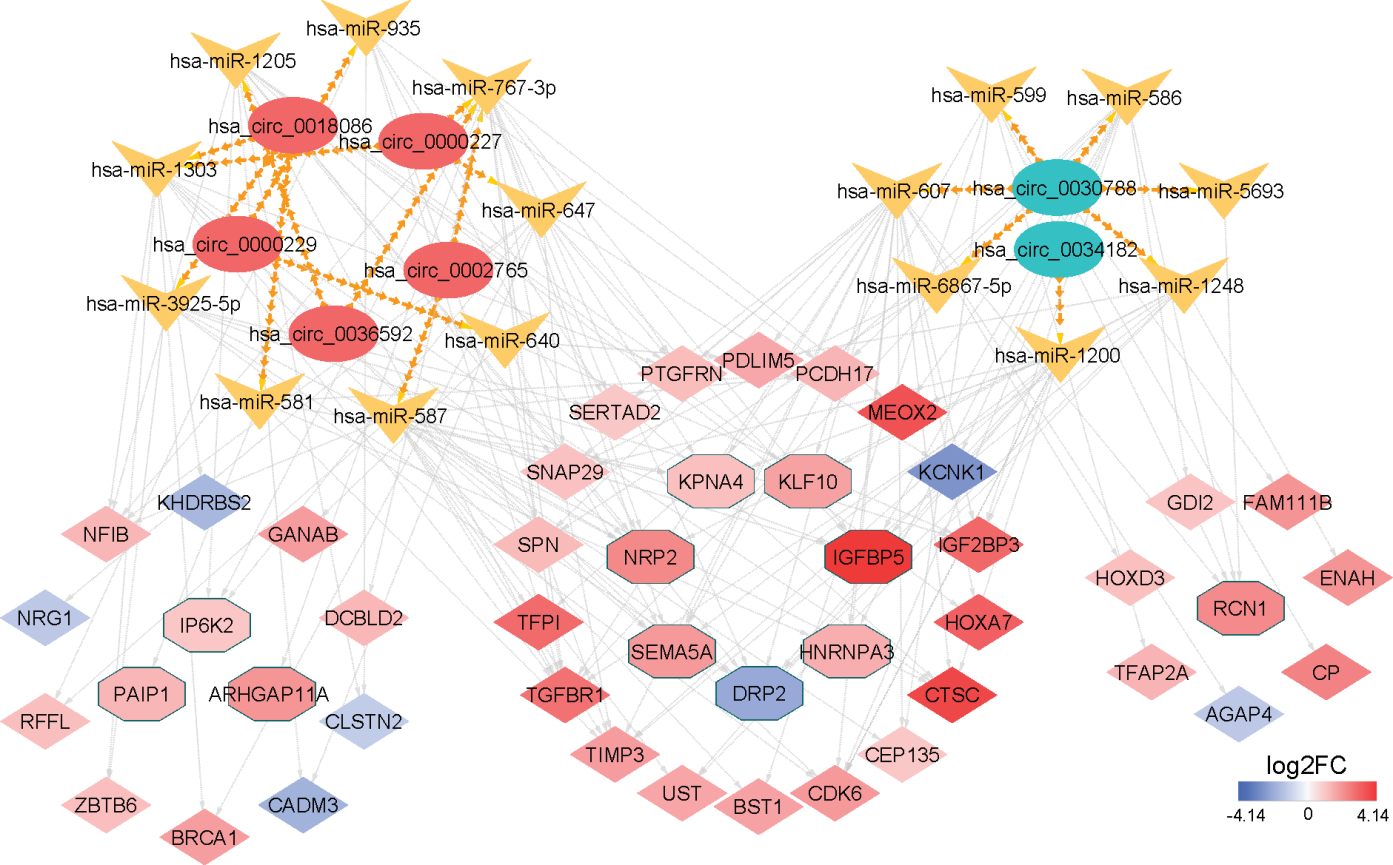
A network of circRNA/miRNA/mRNA in glioma. Oval, arrow, diamond, and octagon represents circRNA, miRNA, mRNA, and hub mRNAs respectively. For circRNAs, red indicates upregulation and blue means downregulation. Gradual color changes of mRNAs represent differences in the expression levels.

To present the relationship between circRNA, miRNA, and mRNA, a ceRNA network consisting of seven circRNAs, 15 miRNAs, and 46 mRNAs was constructed using Cytoscape as shown in Fig. 7. The five upregulated circRNAs influenced nine miRNAs and 13 mRNAs, while the two downregulated circRNAs targeted seven miRNAs and eight mRNAs. Meanwhile, 25 mRNAs were under the two-way regulation of both groups of circRNAs.

### Immune cell infiltration features of prognosis-related genes

**Figure 8:**
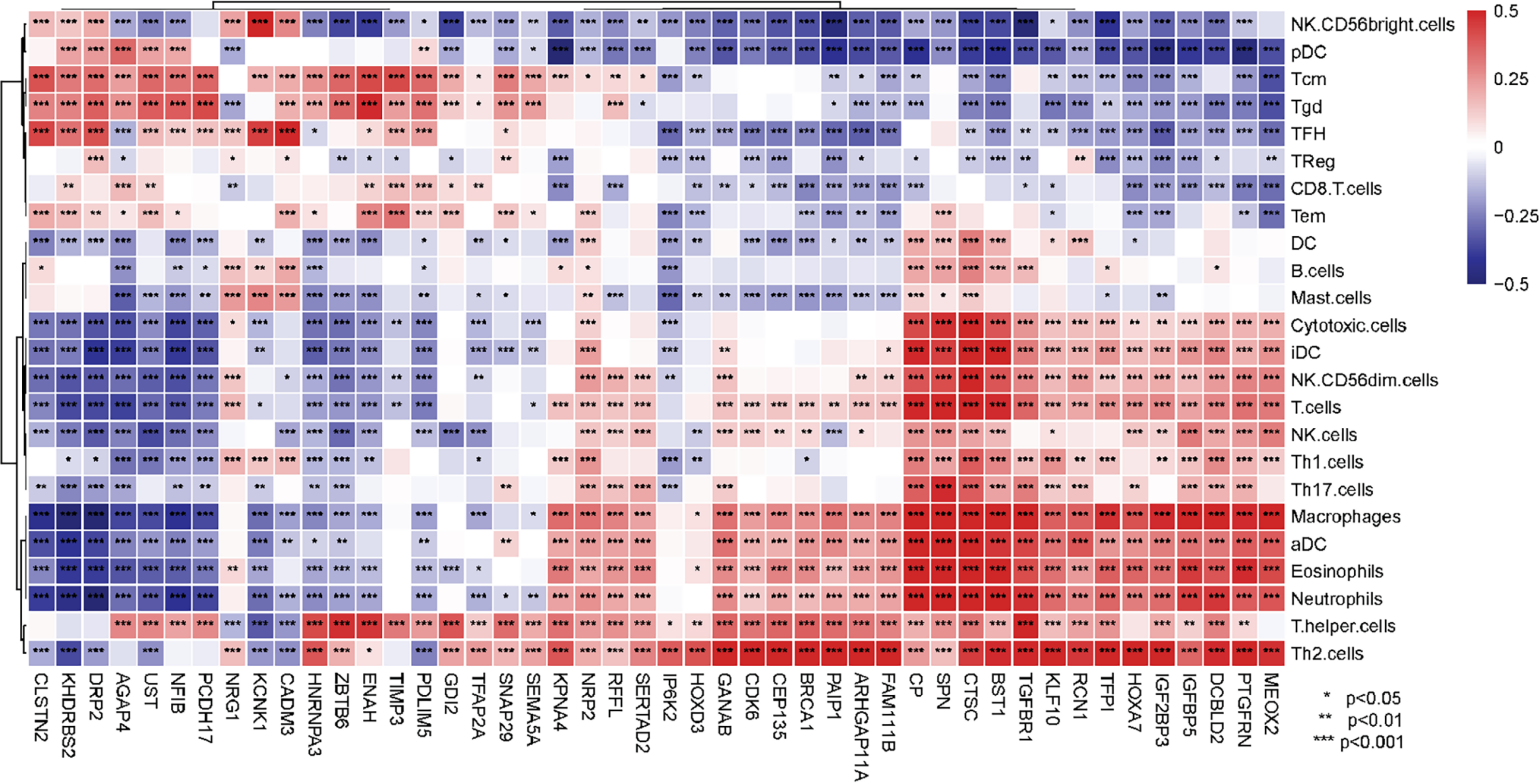
Correlation between 24 immune cells infiltration level and 46 LASSO regression analysis significant genes.

Gene expression profiles from TCGA-GBMLGG dataset and immune cell signatures from a previous study were used for immune infiltration analysis with the GSVA package (Bindea et al., 2013; Hanzelmann, Castelo & Guinney, 2013). Spearman correlation between infiltration levels of 24 immune cells and 46 prognosis-related genes demonstrated that their expression levels were closely related to tumor microenvironment immune infiltration (Fig. 8).

### Candidate compounds from CMap and assessment

**Figure 9:**
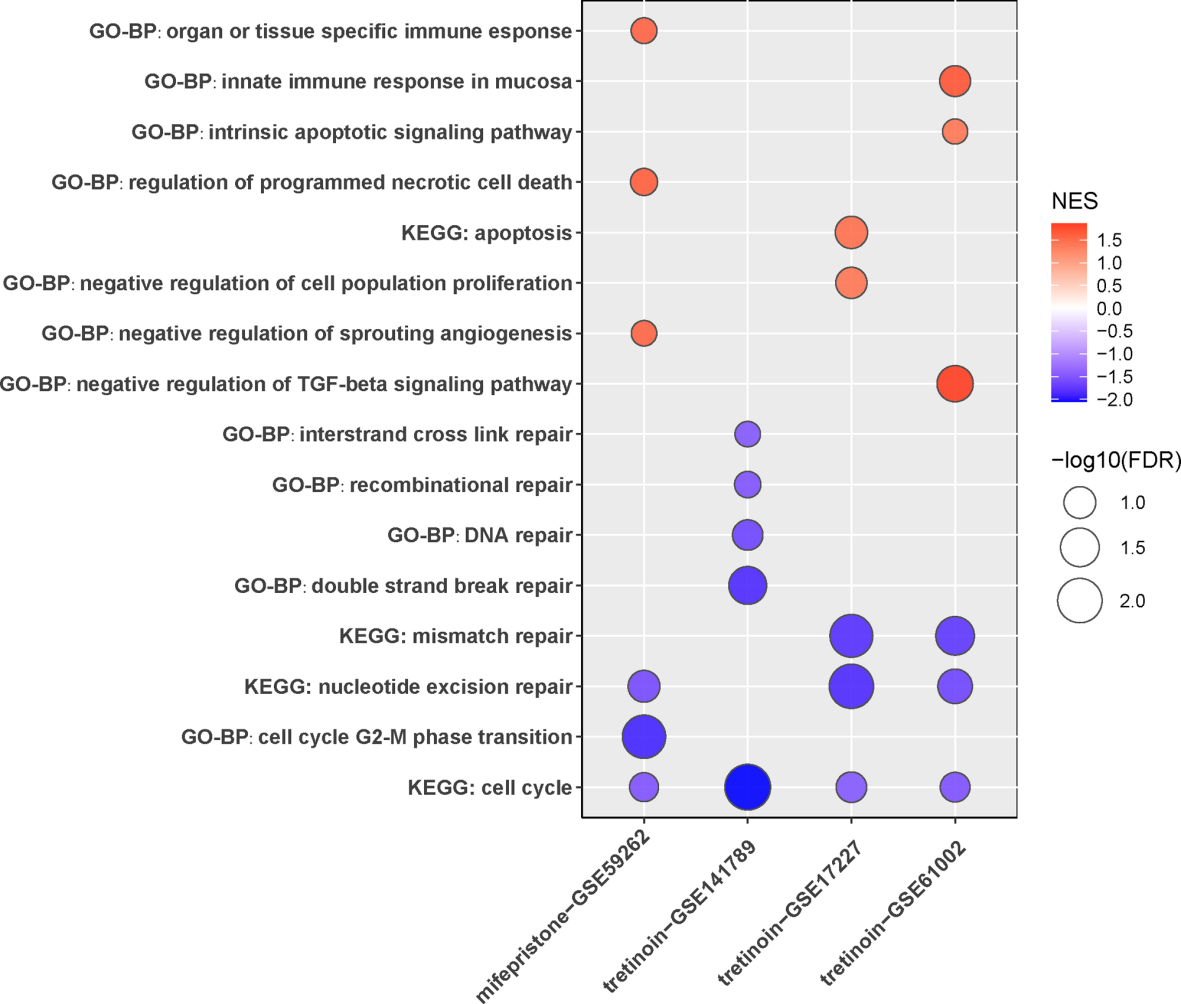
The impact of candidate drugs on glioma cells. mRNA expression profiles of glioma cells treated with drug(mifepristone/tretinoin) or vehicle were analyzed with GSEA. NES, normalized enrichment score; FDR, false discovery rate. Negative value of NES means inhibition; positive value means promotion. FDR <0.25 were considered as significant.

Notably, in order to explore the practical value of this study, the candidate compounds that might have effects on gliomas were predicted by CMap with the hub genes we screened out (Table 3). Based on the enrichment correlation coefficient, drugs such as fulvestrant, tanespimycin, mifepristone, tretinoin, and harman are the most promising potential therapeutic options for gliomas. With GSEA analysis on RNA-seq data from GEO (GSE59262 for mifepristone; GSE141789, GSE17227, and GSE61002 for tretinoin), mifepristone and tretinoin were proven to inhibit cell cycle and DNA repair pathways (Fig. 9). These novel therapeutic options would require more preclinical and clinical studies for further validation.

**Table 3:** Potential therapeutic options forecasted by CMap.

cmap name	dose	cell	score	up	down	instance_id
fulvestrant	10 nM	PC3	−1	−0.212	0.661	4462
tanespimycin	1 µM	PC3	−0.999	−0.407	0.465	1218
mifepristone	9 µM	HL60	−0.983	−0.433	0.425	1569
tretinoin	1 µM	PC3	−0.955	−0.276	0.558	1211
harman	18 µM	PC3	−0.953	−0.495	0.337	4584
miconazole	10 µM	HL60	−0.941	−0.453	0.368	1977
ifosfamide	15 µM	PC3	−0.933	−0.26	0.554	5805
trimethylcolchicinic acid	12 µM	PC3	−0.931	−0.457	0.356	4202
rifampicin	5 µM	PC3	−0.909	−0.281	0.512	4008

## Discussion

CircRNA can act as a molecular sponge for miRNAs to de-repress all target genes of these miRNAs (Kristensen et al., 2019; Meng et al., 2017). Accumulating evidence has shown that this circRNA-miRNA-mRNA network plays an important role in the pathogenesis of gliomas, encompassing a wide range of phenotypes, such as proliferation, migration, and invasion (Chen et al., 2019; Ding et al., 2020; Wang et al., 2018). Therefore, circRNAs and miRNAs are increasingly regarded as promising therapeutic targets or diagnostic biomarkers. GBM can be divided into different subsets with diverse responses to various therapies as a group of heterogeneous intracranial neoplasms with distinct histopathological and molecular biological characteristics (Lapointe, Perry & Butowski, 2018). Thus, there is an urgent need to establish reliable risk stratification methods to classify GBM patients into various risk groups to benefit from various treatment strategies. Many studies have explored the prognostic signatures of gliomas in the context of epigenetic modifications or lncRNAs (Niu et al., 2020; Pan et al., 2021). However, so far, there has been no comprehensive and in-depth study of the molecular signatures of circRNA-related ceRNA networks in gliomas. Therefore, in this study, we constructed a circRNA–miRNA–mRNA network in glioma to help understand its pathogenesis as well as aid in risk stratification and therapeutic decision-making.

In this study, eight DECs (hsa_circ_0001156, hsa_circ_0030788, hsa_circ_0034182, hsa_circ_0000227, hsa_circ_0018086, hsa_circ_0000229, hsa_circ_0036592, and hsa_circ_0002765) were identified as DECs in the first step. To the best of our knowledge, all of them were found to be abnormally expressed in glioma for the first time and have not been studied so far, which makes them potential novel biomarkers or therapeutic targets.

Seven out of eight circRNAs (except for hsa_circ_0001156) were identified as ceRNAs to bind 15 miRNAs (hsa-miR-1200, hsa-miR-1205, hsa-miR-1248, hsa-miR-1303, hsa-miR-3925-5p, hsa-miR-5693, hsa-miR-581, hsa-miR-586, hsa-miR-599, hsa-miR-607, hsa-miR-640, hsa-miR-647, hsa-miR-6867-5p, hsa-miR-767-3p, and hsa-miR-935). As for hsa_circ_0001156, it might still be involved in the pathogenesis of gliomas through functions other than miRNA sponges, such as coding proteins, interacting with RNA binding proteins, or modulating the stability of mRNAs.

As shown in Table 2, among the 15 miRNAs identified, miR-581, miR-586, and miR-1248 were reported to promote tumor progression, while miR-599, miR-607, and miR-767-3p were shown to be tumor suppressors in various cancers. Some studies have shown contradictory results regarding the effects of miR-1303, miR-647, and miR-935 on some tumors. This could partly result from differences in the cell lines used, the phenotypes selected, or the pathways studied. However, further research is needed to resolve these discrepancies. Nevertheless, it is noteworthy that miR-647, miR-767-3p, and miR-935 reportedly suppress the progression of glioma through multiple regulatory axes, including miR-647/HOXA9, miR-935/FZD6, miR-935/HIF1 *α*, or miR-767-3p itself, making them promising biomarkers and therapeutic targets for glioma. In addition, the other six miRNAs without previous studies on tumors (miR-1200, miR-1205, miR-3925-5p, miR-5693, miR-640, and miR-6867-5p) could also be novel fields worth exploring, which could possibly lead to unexpected discoveries.

Given the correlation between miRNAs expression levels and their biological functions, we further checked their expression levels in glioma tissues with CGGA data and GEO datasets, as shown in (Tables S2–S3). According to those two datasets, the miR607 and miR587 have relatively lower expression levels, implying they could be less important in glioma studies. And miR581 has relatively higher expression, giving it higher importance for glioma. But it is noteworthy that different datasets showed considerably different expression of those miRNAs, probably due to the different platforms used and various samples chosen. And their expression values vary quite a lot from sample to sample even for the same dataset. So, their actual expression levels still await to be examined.

CircRNAs fulfill their functions by de-repressing the target genes of miRNAs. Therefore, to further explore the effects of circRNAs on glioma, 1076 overlapping target genes were collected and used for functional enrichment analyses. The ten major hallmarks of tumors include sustaining proliferative signaling, evading growth suppressors, resisting cell death, enabling replicative immortality, inducing angiogenesis, activating invasion and metastasis, reprogramming of energy metabolism, evading immune destruction, genome instability and mutation, and dysregulating cellular energetics (Hanahan & Weinberg, 2011), most of which were covered in our enrichment result of biological processes. This indicates that the ceRNA network we built here is extensively involved in the initiation and progression of gliomas.

The pathway enrichment results uncovered the involvement of many essential tumor-related pathways such as the Wnt, TGFβ, cell cycle, p53, Hippo, MAPK, and stemness regulating pathways. Wnt signaling, commonly divided into *β*-catenin-dependent (canonical) and independent (non-canonical) signaling, is one of the key cascades regulating development (Klaus & Birchmeier, 2008). Its role in carcinogenesis has mostly been described in colorectal cancer, along with some other cancer entities (Zhan, Rindtorff & Boutros, 2017). The transforming growth factor (TGF)-*β* signaling pathway is deregulated in many diseases and has dual functions in cancers. It suppresses tumors in healthy cells and early stage cancer cells but promotes tumorigenesis, metastasis, and chemoresistance in late-stage cancer (Colak & Ten Dijke, 2017). p53 is a tumor suppressor protein that regulates cell growth by promoting apoptosis and DNA repair under stressful conditions (Kanapathipillai, 2018). The Hippo pathway largely consists of a kinase cascade (MST1/2 and LATS1/2) and downstream transcriptional coactivators (YAP and TAZ), controlling transcriptional programs involved in cell proliferation, survival, mobility, stemness, and differentiation (Ma et al., 2019). The MAPK/ERK pathway is a chain of proteins that communicate signals from a receptor on the cell surface to the DNA in the nucleus of the cell (Orton et al., 2005). Alteration of this pathway is often a necessary step in the development of many cancers (Drosten & Barbacid, 2020). Cancer stem cells are capable of sustaining tumors by aiding metastasis, therapy resistance, and tumor microenvironment maintenance, making the stemness regulation key traits and mechanisms for tumor progression (Saygin et al., 2019). All of these pathways, which were under the control of the ceRNA network we constructed in this study, have been shown to participate in the initiation or progression of gliomas (He et al., 2019; Lan et al., 2019; Lee et al., 2017; Ma et al., 2018; Masliantsev, Karayan-Tapon & Guichet, 2021; Zhao et al., 2019). Altogether, these 1076 target genes, regulated indirectly by the circRNAs identified in the present study, play essential roles in the pathogenesis of gliomas.

Thereafter, LASSO regression analysis and multivariate Cox regression analysis were applied to the 1076 genes consecutively. Forty-six LASSO significant genes and 11 independent prognosis-related hub genes were identified (ARHGAP11A, DRP2, HNRNPA3, IGFBP5, IP6K2, KLF10, KPNA4, NRP2, PAIP1, RCN1, and SEMA5A). Among them, ARHGAP11A, DRP2, HNRNPA3, KLF10, PAIP1, and RCN1 have not yet been studied in gliomas. Three mRNAs were identified as oncogenes in gliomas, which was consistent with our multivariate Cox regression analysis result: IGFBP5 can increase cell invasion and inhibit cell proliferation via the EMT and Akt signaling pathways in GBM (Dong et al., 2020); IP6K2 was reported to promote cell proliferation and inhibit cell apoptosis under the regulation of the LINC00467/miR-339-3p axis (Liang & Tang, 2020); and KPNA4 is capable of facilitating epithelial-mesenchymal transition in glioma, which can be suppressed by miR-181b, a tumor-suppressive miRNA (Wang et al., 2015). Surprisingly, the roles of the other two mRNAs in glioma were shown to be different from our analysis result: NRP2 promoted glioma cell growth, invasion, and angiogenesis (Zheng et al., 2013); and SEMA5A, whose expression is markedly reduced in higher grades of glioma, can impede motility and promote differentiation of human gliomas (Li & Lee, 2010). This discrepancy might result from the fact that we used survival data for analysis, and those genes were studied only *in vitro* for some specific phenotypes, while the *in vivo* result of survival might be influenced by multiple other conditions and phenotypes (such as immune response and therapy sensitivity). However, the functions of these genes require further experimental verification. Nevertheless, the K-M curve analysis and ROC curve analysis in both training and external validation datasets proved that these genes together are a competent signature for predicting the prognosis of gliomas.

In addition, we checked the human protein atlas database to confirm the protein expression level changes of the 11 hub genes. Three of them (DRP2, IGFBP5, KLF10) are not provided with protein expression information. Two (ARHGAP11A, NRP2) of them showed no big difference between normal and glioma samples. The other six of them (HNRNPA3, IP6K2, KPNA4, PAIP1, RCN1, SEMA5A) showed significantly increased protein expression, consistent with our results (Fig. S2). This does not mean that DRP2, IGFBP5, KLF10, ARHGAP11A and NRP2 are not essential genes for glioma pathogenesis, given the missing data and limited sample size we found. But still this could give us some hints that those genes with supporting HPA results (namely HNRNPA3, IP6K2, KPNA4, PAIP1, RCN1, SEMA5A) could be more promising targets for future further validation studies.

Recently, circRNAs were reported to play a significant role in multiple immune-related biological processes, including innate and adaptive immune responses, immune cell homeostasis, immune recognition, and anti-tumor immunity (Yan & Chen, 2020; Zhang et al., 2020). Comprehensive recognition of circRNA-mediated immune cell infiltration in glioma can provide novel insights into risk stratification and clinical therapeutic strategies. Hence, we profiled tumor microenvironment immune cell infiltration utilizing 46 prognosis-related genes from the LASSO regression analysis as shown in Fig. 8. The current consensus is that the anti-tumor immune response in glioma is largely suppressed by brain-resident microglial cells and bone marrow-derived macrophages, and is mainly promoted by CD8+ T cells (Pinton et al., 2019). However, the roles of other immune cells, such as B cells, are still debatable (Pinton et al., 2019; VonRoemeling et al., 2020). Our results showed that generally the genes that were positively related to macrophages were negatively correlated with CD8+ T cells, and vice versa. This indicated that these genes could play considerable roles in immune infiltration switch of the tumor microenvironment. However, it should be noted that there is still a lack of systemic immune cell markers for gliomas. Existing immune markers are mostly constructed in other tumors, and some glioma-specific immune cells (such as microglial cells) still lack convincing specific markers. Specific immune cell markers for gliomas are urgently needed for a robust assessment of immune infiltration and understanding of immune response mechanisms in gliomas.

Glioma is one of the most drug-resistant malignancies with frequent recurrence after chemotherapy, making it necessary to explore novel compounds or drugs that may have a therapeutic effect. Here, with the hub genes identified, some potential drugs were acquired from the CMap database. Although the prediction of CMap was mostly based on experiments in prostate cancer and leukemia cell lines, the effects of these drugs have also been verified in multiple other tumors. For instance, fulvestrant is a selective estrogen receptor degrader that has been extensively studied for its therapeutic effects in breast cancer (Slamon et al., 2020). Harmane is a tremorigenic *β*-carboline capable of inhibiting mitochondrial viability and increasing reactive oxygen species levels (Khan, Patel & Kamal, 2017). Its semi-synthetic derivative, B-9-3, showed an anti-proliferative effect in lung cancer, breast cancer, and colorectal carcinoma cell lines via induction of apoptosis and inhibition of cell migration (Daoud et al., 2014). Some of these drugs have been proven to interfere with the progression of gliomas. Tanespimycin is a well-characterized HSP90 inhibitor that can inhibit the growth of GBM and synergize with radiation (Sauvageot et al., 2009). Mifepristone was reported to be a potential therapy for reducing angiogenesis and TMZ resistance in GBM (Llaguno-Munive et al., 2020). Tretinoin, an all-trans retinoic acid, was shown to significantly induce apoptosis and suppress stemness in GBM (Chen et al., 2014; Hu et al., 2017). Importantly, mifepristone and tretinoin were shown to inhibit cell cycle and DNA repair of glioma according to our own GSEA analysis result (Fig. 9). Given that radiation and temozolomide, the major non-surgical treatments for glioma, both work through inducing DNA damage, those novel drugs could be promising supplementary therapeutic treatments which can be applied in combination with radiotherapy or chemotherapy.

Several limitations of this study should be considered. The construction of circRNA/miRNA/mRNA regulatory networks and the prediction of therapeutic drugs largely relied on a series of bioinformatics algorithms and databases, whose authenticity and accuracy still await the verification of numerous experiments. Therefore, we adopted and integrated multiple databases for all predictions in the present study to improve robustness. In addition, the retrospective research design could display some statistical bias and the traditional bulk sequence transcriptome data would lack comprehensive exploration of intra-tumoral heterogeneity. A prospective study design and utilization of single-cell omics techniques will help address this issue and provide more accurate and reliable results in the future. However, based on circRNA/miRNA/mRNA regulatory networks, we established a superior predictive signature to assess the clinical outcomes of patients with GBM and forecasted some promising candidate drugs.

## Conclusions

Through the construction of a circRNA/miRNA/mRNA regulatory network in glioma and the combination of survival analysis, this study successfully identified 11 circRNA-related mRNA signatures to predict the prognosis of GBM patients. Additionally, we determined that circRNA-regulated hub genes were correlated with specific immune cell infiltration levels and proposed some potential therapeutic options. Comprehensively exploring the circRNA/miRNA/mRNA regulatory network in GBM will enhance our understanding of the pathogenesis and immune infiltration features of glioma, promote treatment strategies, and improve clinical outcomes.

##  Supplemental Information

10.7717/peerj.11894/supp-1 Supplemental Information 1 Codes used to generate figures

10.7717/peerj.11894/supp-2 Supplemental Information 2 CirRNA expression level in the datasets studied in this paper

10.7717/peerj.11894/supp-3 Supplemental Information 3 Target miRNA expression level in CCGA 198 samples miRNA seq dataset

10.7717/peerj.11894/supp-4 Supplemental Information 4 GSE165286-NT vs GBM targets micro RNA expression level-FPKM

10.7717/peerj.11894/supp-5 Supplemental Information 5 CGGA sample ID list

10.7717/peerj.11894/supp-6 Supplemental Information 6 TCGA sample ID list

10.7717/peerj.11894/supp-7 Supplemental Information 7 No gender correlation of target mRNAs (A) PCA plot of gene expression level between males and females. (B) volcano plot of differentially expressed genes between males and females.

10.7717/peerj.11894/supp-8 Supplemental Information 8 The Human Protein Atlas images of the 6 hub genes with consistent supporting results

## Additional Information and Declarations

### Data Availability

The following information was supplied regarding data availability: The codes used for bioinformatics analysis are available in the Supplementary File.

## References

[ref-1] Ali HR, Chlon L, Pharoah PD, Markowetz F, Caldas C (2016). Patterns of immune infiltration in breast cancer and their clinical implications: a gene-expression-based retrospective study. PLOS Medicine.

[ref-2] Barrett T, Wilhite SE, Ledoux P, Evangelista C, Kim IF, Tomashevsky M, Marshall KA, Phillippy KH, Sherman PM, Holko M, Yefanov A, Lee H, Zhang N, Robertson CL, Serova N, Davis S, Soboleva A (2013). NCBI GEO: archive for functional genomics data sets–update. Nucleic Acids Research.

[ref-3] Ba Y, Liu Y, Li C, Zhu Y, Xing W (2020). HIPK3 Promotes Growth and Metastasis of Esophageal Squamous Cell Carcinoma via Regulation of miR-599/c-MYC Axis. OncoTargets and Therapy.

[ref-4] Bindea G, Mlecnik B, Tosolini M, Kirilovsky A, Waldner M, Obenauf AC, Angell H, Fredriksen T, Lafontaine L, Berger A, Bruneval P, Fridman WH, Becker C, Pages F, Speicher MR, Trajanoski Z, Galon J (2013). Spatiotemporal dynamics of intratumoral immune cells reveal the immune landscape in human cancer. Immunity.

[ref-5] Binnewies M, Roberts EW, Kersten K, Chan V, Fearon DF, Merad M, Coussens LM, Gabrilovich DI, Ostrand-Rosenberg S, Hedrick CC, Vonderheide RH, Pittet MJ, Jain RK, Zou W, Howcroft TK, Woodhouse EC, Weinberg RA, Krummel MF (2018). Understanding the tumor immune microenvironment (TIME) for effective therapy. Nature Medicine.

[ref-6] Cao W, Wei W, Zhan Z, Xie D, Xie Y, Xiao Q (2017). Role of miR-647 in human gastric cancer suppression. Oncology Reports.

[ref-7] Cao W, Wei W, Zhan Z, Xie D, Xie Y, Xiao Q (2018). Regulation of drug resistance and metastasis of gastric cancer cells via the microRNA647-ANK2 axis. International Journal of Molecular Medicine.

[ref-8] Chen J, Chen T, Zhu Y, Li Y, Zhang Y, Wang Y, Li X, Xie X, Wang J, Huang M, Sun X, Ke Y (2019). circPTN sponges miR-145-5p/miR-330-5p to promote proliferation and stemness in glioma. Journal of Experimental & Clinical Cancer Research.

[ref-9] Chen LL (2020). The expanding regulatory mechanisms and cellular functions of circular RNAs. Nature Reviews Molecular Cell Biology.

[ref-10] Chen W, Cen S, Zhou X, Yang T, Wu K, Zou L, Luo J, Li C, Lv D, Mao X (2020a). Circular RNA CircNOLC1, Upregulated by NF-KappaB, Promotes the Progression of Prostate Cancer via miR-647/PAQR4 Axis. Frontiers in Cell and Developmental Biology.

[ref-11] Chen Y, Du M, Yusuying S, Liu W, Tan Y, Xie P (2020b). Nedd8-activating enzyme inhibitor MLN4924 (Pevonedistat), inhibits miR-1303 to suppress human breast cancer cell proliferation via targeting p27(Kip1). Experimental Cell Research.

[ref-12] Chen PH, Shih CM, Chang WC, Cheng CH, Lin CW, Ho KH, Su PC, Chen KC (2014). MicroRNA-302b-inhibited E2F3 transcription factor is related to all trans retinoic acid-induced glioma cell apoptosis. Journal of Neurochemistry.

[ref-13] Chen Y, Zhao J, Duan Z, Gong T, Chen W, Wang S, Xu H (2019). miR27b3p and miR607 cooperatively regulate BLM gene expression by directly targeting the 3’UTR in PC3 cells. Mol Med Rep.

[ref-14] Colak S, Ten Dijke P (2017). Targeting TGF-beta signaling in cancer. Trends Cancer.

[ref-15] Daoud A, Song J, Xiao F, Shang J (2014). B-9-3, a novel beta-carboline derivative exhibits anti-cancer activity via induction of apoptosis and inhibition of cell migration in vitro. European Journal of Pharmacology.

[ref-16] Ding C, Yi X, Wu X, Bu X, Wang D, Wu Z, Zhang G, Gu J, Kang D (2020). Exosome-mediated transfer of circRNA CircNFIX enhances temozolomide resistance in glioma. Cancer Letters.

[ref-17] Dong C, Zhang J, Fang S, Liu F (2020). IGFBP5 increases cell invasion and inhibits cell proliferation by EMT and Akt signaling pathway in Glioblastoma multiforme cells. Cell Division.

[ref-18] Drosten M, Barbacid M (2020). Targeting the MAPK pathway in KRAS-driven tumors. Cancer Cell.

[ref-19] Dudekula DB, Panda AC, Grammatikakis I, De S, Abdelmohsen K, Gorospe M (2016). CircInteractome: a web tool for exploring circular RNAs and their interacting proteins and microRNAs. RNA Biology.

[ref-20] Dweep H, Gretz N (2015). miRWalk2.0: a comprehensive atlas of microRNA-target interactions. Nature Methods.

[ref-21] Hanahan D, Weinberg RA (2011). Hallmarks of cancer: the next generation. Cell.

[ref-22] Hansen TB, Jensen TI, Clausen BH, Bramsen JB, Finsen B, Damgaard CK, Kjems J (2013). Natural RNA circles function as efficient microRNA sponges. Nature.

[ref-23] Hanzelmann S, Castelo R, Guinney J (2013). GSVA: gene set variation analysis for microarray and RNA-seq data. BMC Bioinformatics.

[ref-24] He L, Zhou H, Zeng Z, Yao H, Jiang W, Qu H (2019). Wnt/beta-catenin signaling cascade: a promising target for glioma therapy. Journal of Cellular Physiology.

[ref-25] Hu PS, Xia QS, Wu F, Li DK, Qi YJ, Hu Y, Wei ZZ, Li SS, Tian NY, Wei QF, Shen LJ, Yin B, Jiang T, Yuan JG, Qiang BQ, Han W, Peng XZ (2017). NSPc1 promotes cancer stem cell self-renewal by repressing the synthesis of all-trans retinoic acid via targeting RDH16 in malignant glioma. Oncogene.

[ref-26] Huang G, Chen J, Liu J, Zhang X, Duan H, Fang Q (2020). MiR-935/HIF1alpha Feedback Loop Inhibits the Proliferation and Invasiveness of Glioma. OncoTargets and Therapy.

[ref-27] Jiang W, Wen D, Gong L, Wang Y, Liu Z, Yin F (2018). Circular RNA hsa_circ_0000673 promotes hepatocellular carcinoma malignance by decreasing miR-767-3p targeting SET. Biochemical and Biophysical Research Communications.

[ref-28] Jiang W, Zhao X, Yang W (2021). MiR-647 promotes cisplatin-induced cell apoptosis via downregulating IGF2 in non-small cell lung cancer. Minerva Medica.

[ref-29] Kanapathipillai M (2018). Treating p53 mutant aggregation-associated cancer. Cancers.

[ref-30] Khan H, Patel S, Kamal MA (2017). Pharmacological and toxicological profile of harmane-beta-carboline alkaloid: friend or foe. Current Drug Metabolism.

[ref-31] Klaus A, Birchmeier W (2008). Wnt signalling and its impact on development and cancer. Nature Reviews Cancer.

[ref-32] Kreth S, Limbeck E, Hinske LC, Schutz SV, Thon N, Hoefig K, Egensperger R, Kreth FW (2013). In human glioblastomas transcript elongation by alternative polyadenylation and miRNA targeting is a potent mechanism of MGMT silencing. Acta Neuropathologica.

[ref-33] Kristensen LS, Andersen MS, Stagsted LVW, Ebbesen KK, Hansen TB, Kjems J (2019). The biogenesis, biology and characterization of circular RNAs. Nature Reviews Genetics.

[ref-34] Lamb J (2007). The Connectivity Map: a new tool for biomedical research. Nature Reviews Cancer.

[ref-35] Lamb J, Crawford ED, Peck D, Modell JW, Blat IC, Wrobel MJ, Lerner J, Brunet JP, Subramanian A, Ross KN, Reich M, Hieronymus H, Wei G, Armstrong SA, Haggarty SJ, Clemons PA, Wei R, Carr SA, Lander ES, Golub TR (2006). The Connectivity Map: using gene-expression signatures to connect small molecules, genes, and disease. Science.

[ref-36] Lan YL, Zou YJ, Lou JC, Xing JS, Wang X, Zou S, Ma BB, Ding Y, Zhang B (2019). The sodium pump alpha1 subunit regulates bufalin sensitivity of human glioblastoma cells through the p53 signaling pathway. Cell Biology and Toxicology.

[ref-37] Lapointe S, Perry A, Butowski NA (2018). Primary brain tumours in adults. Lancet.

[ref-38] Lee KH, Chen CL, Lee YC, Kao TJ, Chen KY, Fang CY, Chang WC, Chiang YH, Huang CC (2017). Znf179 induces differentiation and growth arrest of human primary glioblastoma multiforme in a p53-dependent cell cycle pathway. Science Reports.

[ref-39] Li X, Lee AY (2010). Semaphorin 5A and plexin-B3 inhibit human glioma cell motility through RhoGDIalpha-mediated inactivation of Rac1 GTPase. Journal of Biological Chemistry.

[ref-40] Li C, Tian Y, Liang Y, Li Q (2020). Circ_0008035 contributes to cell proliferation and inhibits apoptosis and ferroptosis in gastric cancer via miR-599/EIF4A1 axis. Cancer Cell International.

[ref-41] Li L, Guo L, Yin G, Yu G, Zhao Y, Pan Y (2019). Upregulation of circular RNA circ_0001721 predicts unfavorable prognosis in osteosarcoma and facilitates cell progression via sponging miR-569 and miR-599. Biomedicine & Pharmacotherapy.

[ref-42] Li Z, Xu Z, Xie Q, Gao W, Xie J, Zhou L (2016). miR-1303 promotes the proliferation of neuroblastoma cell SH-SY5Y by targeting GSK3beta and SFRP1. Biomedicine & Pharmacotherapy.

[ref-43] Liang R, Tang Y (2020). LINC00467 knockdown repressed cell proliferation but stimulated cell apoptosis in glioblastoma via miR-339-3p/IP6K2 axis. Cancer Biomark.

[ref-44] Liang Y, Song X, Li Y, Chen B, Zhao W, Wang L, Zhang H, Liu Y, Han D, Zhang N, Ma T,  Wang Y, Ye F, Luo D, Li X, Yang Q (2020). LncRNA BCRT1 promotes breast cancer progression by targeting miR-1303/PTBP3 axis. Molecular Cancer.

[ref-45] Liu S, Qu D, Li W, He C, Li S, Wu G, Zhao Q, Shen L, Zhang J, Zheng J (2017a). miR647 and miR1914 promote cancer progression equivalently by downregulating nuclear factor IX in colorectal cancer. Molecular Medicine Reports.

[ref-46] Liu X, Li J, Yu Z, Li J, Sun R, Kan Q (2017b). miR-935 Promotes Liver Cancer Cell Proliferation and Migration by Targeting SOX7. Oncology Research.

[ref-47] Liu Z, Li Q, Zhao X, Cui B, Zhang L, Wang Q (2018). MicroRNA-935 Inhibits Proliferation and Invasion of Osteosarcoma Cells by Directly Targeting High Mobility Group Box 1. Oncology Research.

[ref-48] Liu M, Wang Q, Shen J, Yang BB, Ding X (2019). Circbank: a comprehensive database for circRNA with standard nomenclature. RNA Biology.

[ref-49] Llaguno-Munive M, Leon-Zetina S, Vazquez-Lopez I, Ramos-Godinez MDP, Medina LA, Garcia-Lopez P (2020). Mifepristone as a potential therapy to reduce angiogenesis and p-glycoprotein associated with glioblastoma resistance to temozolomide. Frontiers in Oncology.

[ref-50] Lou C, Zhao J, Gu Y, Li Q, Tang S, Wu Y, Tang J, Zhang C, Li Z, Zhang Y (2020). LINC01559 accelerates pancreatic cancer cell proliferation and migration through YAP-mediated pathway. Journal of Cellular Physiology.

[ref-51] Ma Q, Long W, Xing C, Chu J, Luo M, Wang HY, Liu Q, Wang RF (2018). Cancer stem cells and immunosuppressive microenvironment in glioma. Frontiers in Immunology.

[ref-52] Ma S, Meng Z, Chen R, Guan KL (2019). The hippo pathway: biology and pathophysiology. Annual Review of Biochemistry.

[ref-53] Masliantsev K, Karayan-Tapon L, Guichet PO (2021). Hippo signaling pathway in gliomas. Cells.

[ref-54] Meng S, Zhou H, Feng Z, Xu Z, Tang Y, Li P, Wu M (2017). CircRNA: functions and properties of a novel potential biomarker for cancer. Molecular Cancer.

[ref-55] Niu X, Sun J, Meng L, Fang T, Zhang T, Jiang J, Li H (2020). A Five-lncRNAs signature-derived risk score based on TCGA and CGGA for glioblastoma: potential prospects for treatment evaluation and prognostic prediction. Frontiers in Oncology.

[ref-56] Orton RJ, Sturm OE, Vyshemirsky V, Calder M, Gilbert DR, Kolch W (2005). Computational modelling of the receptor-tyrosine-kinase-activated MAPK pathway. Biochemical Journal.

[ref-57] Ostrom QT, Cote DJ, Ascha M, Kruchko C, Barnholtz-Sloan JS (2018a). Adult glioma incidence and survival by race or ethnicity in the United States from 2000 to 2014. JAMA Oncology.

[ref-58] Ostrom QT, Gittleman H, Truitt G, Boscia A, Kruchko C, Barnholtz-Sloan JS (2018b). CBTRUS statistical report: primary brain and other central nervous system tumors diagnosed in the United States in 2011–2015. Neuro Oncology.

[ref-59] Pan Y, Xiao K, Li Y, Li Y, Liu Q (2021). RNA N6-methyladenosine regulator-mediated methylation modifications pattern and immune infiltration features in glioblastoma. Frontiers in Oncology.

[ref-60] Peng B, Li C, Cai P, Yu L, Zhao B, Chen G (2018). Knockdown of miR935 increases paclitaxel sensitivity via regulation of SOX7 in nonsmallcell lung cancer. Molecular Medicine Reports.

[ref-61] Pinton L, Masetto E, Vettore M, Solito S, Magri S, D’Andolfi M, Bianco PDel, Lollo G, Benoit JP, Okada H, Diaz A, Della Puppa A, Mandruzzato S (2019). The immune suppressive microenvironment of human gliomas depends on the accumulation of bone marrow-derived macrophages in the center of the lesion. Journal for ImmunoTherapy of Cancer.

[ref-62] Qiao G, Wang HB, Duan XN, Yan XF (2021). The effect and mechanism of miR-607/CANT1 axis in lung squamous carcinoma. Anticancer Drugs.

[ref-63] Qin K, Tian G, Chen G, Zhou D, Tang K (2020). miR-647 inhibits glioma cell proliferation, colony formation and invasion by regulating HOXA9. Journal of Gene Medicine.

[ref-64] Quail DF, Joyce JA (2013). Microenvironmental regulation of tumor progression and metastasis. Nature Medicine.

[ref-65] Roemeling CAvon, Wang Y, Qie Y, Yuan H, Zhao H, Liu X, Yang Z, Yang M, Deng W, Bruno KA, Chan CK, Lee AS, Rosenfeld SS, Yun K, Johnson AJ, Mitchell DA, Jiang W, Kim BYS (2020). Therapeutic modulation of phagocytosis in glioblastoma can activate both innate and adaptive antitumour immunity. Nature Communications.

[ref-66] Sauvageot CM, Weatherbee JL, Kesari S, Winters SE, Barnes J, Dellagatta J, Ramakrishna NR, Stiles CD, Kung AL, Kieran MW, Wen PY (2009). Efficacy of the HSP90 inhibitor 17-AAG in human glioma cell lines and tumorigenic glioma stem cells. Neuro Oncology.

[ref-67] Saygin C, Matei D, Majeti R, Reizes O, Lathia JD (2019). Targeting cancer stemness in the clinic: from hype to hope. Cell Stem Cell.

[ref-68] Shannon P, Markiel A, Ozier O, Baliga NS, Wang JT, Ramage D, Amin N, Schwikowski B, Ideker T (2003). Cytoscape: a software environment for integrated models of biomolecular interaction networks. Genome Research.

[ref-69] Slamon DJ, Neven P, Chia S, Fasching PA, De Laurentiis M, Im SA, Petrakova K, Bianchi GV, Esteva FJ, Martin M, Nusch A, Sonke GS, Cruz-Merino LDela, Beck JT, Pivot X, Sondhi M, Wang Y, Chakravartty A, Rodriguez-Lorenc K, Taran T, Jerusalem G (2020). Overall survival with ribociclib plus fulvestrant in advanced breast cancer. New England Journal of Medicine.

[ref-70] Song X, Bi Y, Guo W (2019). Long noncoding RNA PROX1-AS1 promotes tumor progression and aggressiveness by sponging miR-647 in gastric cancer. Minerva Medica.

[ref-71] Sun D, Liu J, Zhou L (2019). Upregulation of circular RNA circFAM53B predicts adverse prognosis and accelerates the progression of ovarian cancer via the miR646/VAMP2 and miR647/MDM2 signaling pathways. Oncology Reports.

[ref-72] Sun D, Zhu D (2020). Circular RNA hsa_circ_0001649 suppresses the growth of osteosarcoma cells via sponging multiple miRNAs. Cell Cycle.

[ref-73] Szklarczyk D, Gable AL, Lyon D, Junge A, Wyder S, Huerta-Cepas J, Simonovic M, Doncheva NT, Morris JH, Bork P, Jensen LJ, Mering CV (2019). STRING v11: protein-protein association networks with increased coverage, supporting functional discovery in genome-wide experimental datasets. Nucleic Acids Research.

[ref-74] Tan X, Wang P, Lou J, Zhao J (2020). Knockdown of lncRNA NEAT1 suppresses hypoxia-induced migration, invasion and glycolysis in anaplastic thyroid carcinoma cells through regulation of miR-206 and miR-599. Cancer Cell International.

[ref-75] Tang Z, Kang B, Li C, Chen T, Zhang Z (2019). GEPIA2: an enhanced web server for large-scale expression profiling and interactive analysis. Nucleic Acids Research.

[ref-76] Tian J, Hu X, Gao W, Zhang J, Chen M, Zhang X, Ma J, Yuan H (2016). Identification a novel tumor-suppressive hsa-miR-599 regulates cells proliferation. migration and invasion by targeting oncogenic MYC in hepatocellular carcinoma. American Journal of Translational Research.

[ref-77] Tokar T, Pastrello C, Rossos AEM, Abovsky M, Hauschild AC, Tsay M, Lu R, Jurisica I (2018). mirDIP 4.1-integrative database of human microRNA target predictions. Nucleic Acids Research.

[ref-78] Uhlen M, Fagerberg L, Hallstrom BM, Lindskog C, Oksvold P, Mardinoglu A, Sivertsson A, Kampf C, Sjostedt E, Asplund A, Olsson I, Edlund K, Lundberg E, Navani S, Szigyarto CA, Odeberg J, Djureinovic D, Takanen JO, Hober S, Alm T, Edqvist PH, Berling H, Tegel H, Mulder J, Rockberg J, Nilsson P, Schwenk JM, Hamsten M, Feilitzen Kvon, Forsberg M, Persson L, Johansson F, Zwahlen M, Von Heijne G, Nielsen J, Ponten F (2015). Proteomics, tissue-based map of the human proteome. Science.

[ref-79] Vlachos IS, Zagganas K, Paraskevopoulou MD, Georgakilas G, Karagkouni D, Vergoulis T, Dalamagas T, Hatzigeorgiou AG (2015). DIANA-miRPath v3.0: deciphering microRNA function with experimental support. Nucleic Acids Research.

[ref-80] Wan YL, Dai HJ, Liu W, Ma HT (2018). miR-767-3p Inhibits Growth and Migration of Lung Adenocarcinoma Cells by Regulating CLDN18. Oncology Research.

[ref-81] Wang C, Li S, Xu J, Niu W, Li S (2019a). microRNA-935 is reduced in non-small cell lung cancer tissue. is linked to poor outcome, and acts on signal transduction mediator E2F7 and the AKT pathway. British Journal of Biomedical Science.

[ref-82] Wang C, Tang D, Wang H, Hu G, Hu S, Li L, Min B, Wang Y (2019b). Circular RNA hsa_circ_0030018 acts as a sponge of miR-599 to aggravate esophageal carcinoma progression by regulating ENAH expression. Journal of Cellular Biochemistry.

[ref-83] Wang DP, Tang XZ, Liang QK, Zeng XJ, Yang JB, Xu J (2020). microRNA-599 promotes apoptosis and represses proliferation and epithelial-mesenchymal transition of papillary thyroid carcinoma cells via downregulation of Hey2-depentent Notch signaling pathway. Journal of Cellular Physiology.

[ref-84] Wang X, Jin Y, Zhang H, Huang X, Zhang Y, Zhu J (2018). MicroRNA-599 inhibits metastasis and epithelial-mesenchymal transition via targeting EIF5A2 in gastric cancer. Biomedicine & Pharmacotherapy.

[ref-85] Wang YQ, Ren YF, Song YJ, Xue YF, Zhang XJ, Cao ST, Deng ZJ, Wu J, Chen L, Li G, Shi KQ, Chen YP, Ren H, Huang AL, Tang KF (2014). MicroRNA-581 promotes hepatitis B virus surface antigen expression by targeting Dicer and EDEM1. Carcinogenesis.

[ref-86] Wang H, Tao T, Yan W, Feng Y, Wang Y, Cai J, You Y, Jiang T, Jiang C (2015). Upregulation of miR-181s reverses mesenchymal transition by targeting KPNA4 in glioblastoma. Science Reports.

[ref-87] Wang R, Zhang S, Chen X, Li N, Li J, Jia R, Pan Y, Liang H (2018). EIF4A3-induced circular RNA MMP9 (circMMP9) acts as a sponge of miR-124 and promotes glioblastoma multiforme cell tumorigenesis. Molecular Cancer.

[ref-88] Wesseling P, Capper D (2018). WHO 2016 classification of gliomas. Neuropathology and Applied Neurobiology.

[ref-89] Wu P, Mo Y, Peng M, Tang T, Zhong Y, Deng X, Xiong F, Guo C, Wu X, Li Y, Li X, Li G, Zeng Z, Xiong W (2020). Emerging role of tumor-related functional peptides encoded by lncRNA and circRNA. Molecular Cancer.

[ref-90] Xia S, Feng J, Chen K, Ma Y, Gong J, Cai F, Jin Y, Gao Y, Xia L, Chang H, Wei L, Han L, He C (2018). CSCD: a database for cancer-specific circular RNAs. Nucleic Acids Research.

[ref-91] Xia L, Wu L, Bao J, Li Q, Chen X, Xia H, Xia R (2018). Circular RNA circ-CBFB promotes proliferation and inhibits apoptosis in chronic lymphocytic leukemia through regulating miR-607/FZD3/Wnt/beta-catenin pathway. Biochemical and Biophysical Research Communications.

[ref-92] Xu J, Tian S, Yin Z, Wu S, Liu L, Qian Y, Pei D, Gao W, Xu J, Yin Y, Liu P, Shu Y (2014). MicroRNA-binding site SNPs in deregulated genes are associated with clinical outcome of non-small cell lung cancer. Lung Cancer.

[ref-93] Xu X, Zhou X, Gao C, Cui Y (2020). Hsa_circ_0018818 knockdown suppresses tumorigenesis in non-small cell lung cancer by sponging miR-767-3p. Aging (Albany NY).

[ref-94] Yan L, Chen YG (2020). Circular RNAs in immune response and viral infection. Trends in Biochemical Science.

[ref-95] Yan C, Yu J, Kang W, Liu Y, Ma Z, Zhou L (2016). miR-935 suppresses gastric signet ring cell carcinoma tumorigenesis by targeting Notch1 expression.Biochemical and Biophysical Research Communications.

[ref-96] Yang L, Liu ZM, Rao YW, Cui SQ, Wang H, Jia XJ (2015). Downregulation of microRNA-586 Inhibits Proliferation. Invasion and Metastasis and Promotes Apoptosis in Human Osteosarcoma U2-OS Cell Line. Cytogenet Genome Research.

[ref-97] Yang M, Cui G, Ding M, Yang W, Liu Y, Dai D, Chen L (2016). miR-935 promotes gastric cancer cell proliferation by targeting SOX7. Biomedicine & Pharmacotherapy.

[ref-98] Yang Z, Ma J, Han S, Li X, Guo H, Liu D (2020). ZFAS1 Exerts an Oncogenic Role via Suppressing miR-647 in an m(6)A-Dependent Manner in Cervical Cancer. OncoTargets and Therapy.

[ref-99] Ye G, Huang K, Yu J, Zhao L, Zhu X, Yang Q, Li W, Jiang Y, Zhuang B, Liu H, Shen Z, Wang D, Yan L, Zhang L, Zhou H, Hu Y, Deng H, Liu H, Li G, Qi X (2017). MicroRNA-647 Targets SRF-MYH9 Axis to Suppress Invasion and Metastasis of Gastric Cancer. Theranostics.

[ref-100] Zhang D, Ma S, Zhang C, Li P, Mao B, Guan X, Zhou W, Peng J, Wang X, Li S, Jia W (2021). MicroRNA-935 Directly Targets FZD6 to Inhibit the Proliferation of Human Glioblastoma and Correlate to Glioma Malignancy and Prognosis. Frontiers in Oncology.

[ref-101] Zhang H, Xue B, Wang S, Li X, Fan T (2019). Long noncoding RNA TP73 antisense RNA 1 facilitates the proliferation and migration of cervical cancer cells via regulating microRNA607/cyclin D2. Molecular Medicine Reports.

[ref-102] Zhang P, Cao L, Fan P, Mei Y, Wu M (2016). LncRNA-MIF. a c-Myc-activated long non-coding RNA, suppresses glycolysis by promoting Fbxw7-mediated c-Myc degradation. EMBO Reports.

[ref-103] Zhang SJ, Feng JF, Wang L, Guo W, Du YW, Ming L, Zhao GQ (2014). miR-1303 targets claudin-18 gene to modulate proliferation and invasion of gastric cancer cells. Digestive Diseases and Sciences.

[ref-104] Zhang X, Zhang M, Wang G, Tian Y, He X (2018a). Tumor promoter role of miR647 in gastric cancer via repression of TP73. Molecular Medicine Reports.

[ref-105] Zhang YS, Chen T, Cai YJ, Dong J, Bai F, Gao X, Tian L, Duan N, Liu D (2018b). MicroRNA-647 promotes the therapeutic effectiveness of argon-helium cryoablation and inhibits cell proliferation through targeting TRAF2 via the NF-kappaB signaling pathway in non-small cell lung cancer. OncoTargets and Therapy.

[ref-106] Zhao X, Liu S, Yan B, Yang J, Chen E (2020). MiR-581/SMAD7 Axis Contributes to Colorectal Cancer Metastasis: A Bioinformatic and Experimental Validation-Based Study. International Journal of Molecular Sciences.

[ref-107] Zhan T, Rindtorff N, Boutros M (2017). Wnt signaling in cancer. Oncogene.

[ref-108] Zhang Q, Wang W, Zhou Q, Chen C, Yuan W, Liu J, Li X, Sun Z (2020). Roles of circRNAs in the tumour microenvironment. Molecular Cancer.

[ref-109] Zhao K, Cui X, Wang Q, Fang C, Tan Y, Wang Y, Yi K, Yang C, You H, Shang R, Wang J, Kang C (2019). RUNX1 contributes to the mesenchymal subtype of glioblastoma in a TGFbeta pathway-dependent manner. Cell Death & Disease.

[ref-110] Zhao Z, Zhang KN, Wang Q, Li G, Zeng F, Zhang Y, Wu F, Chai R, Wang Z, Zhang C, Zhang W, Bao Z, Jiang T (2021). Chinese Glioma Genome Atlas (CGGA): a comprehensive resource with functional genomic data from Chinese gliomas. Genomics Proteomics Bioinformatics.

[ref-111] Zheng Y, Chen Z, Zhou Z, Xu X, Yang H (2020). Silencing of Long Non-Coding RNA LINC00607 Prevents Tumor Proliferation of Osteosarcoma by Acting as a Sponge of miR-607 to Downregulate E2F6. Frontiers in Oncology.

[ref-112] Zheng X, Chopp M, Lu Y, Buller B, Jiang F (2013). MiR-15b and miR-152 reduce glioma cell invasion and angiogenesis via NRP-2 and MMP-3. Cancer Letters.

[ref-113] Zhu Z, Shen S, Zhao S, Wang Z (2020). Hsa_circ_0006916 Knockdown Represses the Development of Hepatocellular Carcinoma via Modulating miR-599/SRSF2 Axis. OncoTargets and Therapy.

