# Supplementary figures and images for: Identification of a circRNA-miRNA-mRNA regulatory network for exploring novel therapeutic options for glioma
Source: PeerJ. 2021 Aug 6;9:e11894. doi: 10.7717/peerj.11894 (PMC8351580; doi:10.7717/peerj.11894)

A

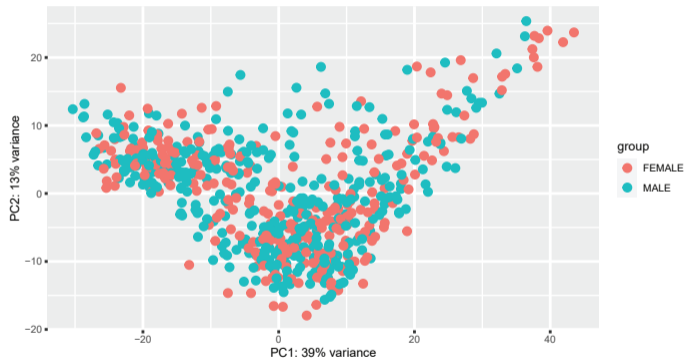

B

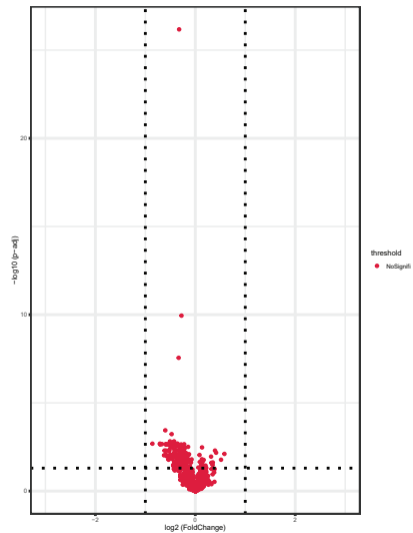

Supplement: Supplemental Information 7 — (A) PCA plot of gene expression level between males and females. (B) volcano plot of differentially expressed genes between males and females. [file peerj-09-11894-s007.pdf]

NT

Glioma

HNRNPA3

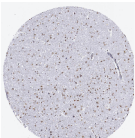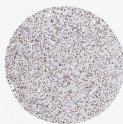

IP3K2

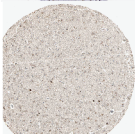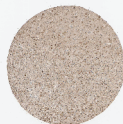

KPNA4

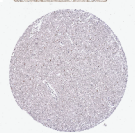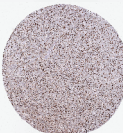

PAIP1

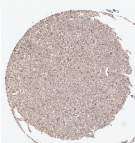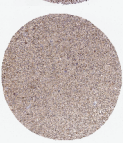

RCN1

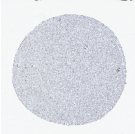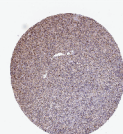

SEMA5A

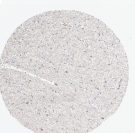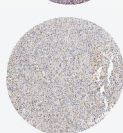

Supplement: Supplemental Information 8 [file peerj-09-11894-s008.pdf]
